# Supplementary material for: Mapping odorant sensitivities reveals a sparse but structured representation of olfactory chemical space by sensory input to the mouse olfactory bulb
Source: eLife. 2022 Jul 21;11:e80470. doi: 10.7554/eLife.80470 (PMC9352350; doi:10.7554/eLife.80470)
Supplement: Supplementary file 1. — Code: abbreviated identifier code, used in Figure 5A and Supplementary file 2. Epifl. est. conc.: estimated delivered concentration of odorant vapor, in mols/L (M), for 1 x dataset. Most commonly-presented values (of 4 preparations) are shown, reported to one significant digit precision. Dilution: liquid dilution of odorant used to generate delivered concentration. Two-photon rel. conc.: concentration used for the two-photon imaging dataset relative to the concentration used in the widefield epifluorescence dataset. Class: nominal odorant classification based on structural features. Odorants in italics gave no response at the given concentration in any of the 8 OBs. [file elife-80470-supp1.docx]

| **Odorant** | **Code** | **CAS#** | **Epifl.**  **est. conc.**  **(M)** | **Dilution** | **Two-photon**  **rel. conc.** | **Class** |
| --- | --- | --- | --- | --- | --- | --- |
| propionic acid | C1 | 79-09-4 | 2E-11 | 1E-05 | 10× | acid |
| butyric acid | C2 | 107-92-6 | 2E-11 | 1E-05 | 10× | acid |
| 2-methylbutyric acid | C3 | 116-53-0 | 6E-12 | 1E-05 | 10× | acid |
| valeric acid | C4 | 109-52-4 | 6E-12 | 1E-05 | 10× | acid |
| isovaleric acid | C5 | 503-74-2 | 9E-12 | 1E-05 | 10× | acid |
| 2-methyl-2-pentenoic acid | C6 | 3142-72-1 | 4E-14 | 1E-06 | 10× | acid |
| hexanoic acid | C7 | 142-62-1 | 2E-11 | 1E-04 | 10× | acid |
| heptanoic acid | C8 | 111-14-8 | 9E-11 | 1E-03 | 10× | acid |
| *methacrolein* | *D1* | *78-85-3* | *1E-9* | *1E-05* | *10×* | *aldehyde* |
| butyraldehyde | D2 | 123-72-8 | 6E-09 | 1E-04 | 10× | aldehyde |
| isobutyraldehyde | D3 | 78-84-2 | 1E-09 | 1E-05 | 10× | aldehyde |
| 2-methylbutyraldehyde | D4 | 96-17-3 | 8E-11 | 1E-05 | 10× | aldehyde |
| trans-2-methyl-2-butenal | D5 | 497-03-0 | 1E-11 | 1E-06 | 10× | aldehyde |
| valeraldehyde | D6 | 110-62-3 | 2E-09 | 1E-04 | 10× | aldehyde |
| isovaleraldehyde | D7 | 590-86-3 | 4E-09 | 1E-04 | 1× | aldehyde |
| 2-methylvaleraldehyde | D8 | 123-15-9 | 1E-10 | 1E-05 | 10× | aldehyde |
| 2-methyl-2-pentenal | D9 | 623-36-9 | 6E-11 | 1E-05 | 1× | aldehyde |
| hexanal | D10 | 66-25-1 | 7E-10 | 1E-04 | 10× | aldehyde |
| heptanal | D11 | 111-71-7 | 3E-09 | 1E-03 | 10× | aldehyde |
| octanal | D12 | 124-13-0 | 1E-09 | 1E-03 | 10× | aldehyde |
| trans-2-nonenal | D13 | 18829-56-6 | 2E-09 | 1E-02 | 10× | aldehyde |
| trans-2,cis-6-nonadienal | D14 | 557-48-2 | 2E-09 | 1E-02 | 10× | aldehyde |
| trans-2-dodecenal | D15 | 20407-84-5 | 8E-10 | 1E-01 | 10× | aldehyde |
| 2-hexyl-2-decenal | D16 | 13893-39-5 | 5E-09 | 1E-01 | 10× | aldehyde |
| butyl acetate | E1 | 123-86-4 | 9E-10 | 1E-04 | 10× | ester |
| S-methyl thiobutanoate | E2.M1 | 2432-51-1 | 2E-10 | 1E-04 | 10× | mixed |
| isoamyl acetate | E3 | 123-92-2 | 4E-10 | 1E-04 | 10× | ester |
| hexyl acetate | E4 | 142-92-7 | 1E-09 | 1E-03 | 10× | ester |
| ethyl butyrate | E5 | 105-54-4 | 1E-09 | 1E-04 | 10× | ester |
| methyl 2-methylbutyrate | E6 | 868-57-5 | 2E-10 | 1E-05 | 10× | ester |
| vinyl butyrate | E7 | 123-20-6 | 9E-11 | 1E-05 | 10× | ester |
| methyl valerate | E8 | 624-24-8 | 8E-10 | 1E-04 | 10× | ester |
| 1-octen-3-yl butyrate | E9 | 16491-54-6 | 5E-09 | 1E-01 | 10× | ester |
| methyl tiglate | E10 | 6622-76-0 | 1E-11 | 1E-06 | 10× | ester |
| ethyl tiglate | E11 | 5837-78-5 | 2E-12 | 1E-06 | 10× | ester |
| isopropyl tiglate | E12 | 1733-25-1 | 2E-11 | 1E-05 | 10× | ester |
| hexyl tiglate | E13 | 16930-96-4 | 4E-10 | 1E-02 | 10× | ester |
| diacetyl | K1 | 431-03-8 | 4E-09 | 1E-04 | 10× | ketone |
| *2-butanone* | *K2* | *78-93-3* | *7E-10* | *1E-05* | *10×* | *ketone* |
| *2-pentanone* | *K3* | *107-87-9* | *3E-09* | *1E-04* | *10×* | *ketone* |
| 4-methyl-3-penten-2-one | K4 | 141-79-7 | 9.E-10 | 1E-04 | 10× | ketone |
| 2-hexanone | K5 | 591-78-6 | 1E-09 | 1E-04 | 10× | ketone |
| 3-hepten-2-one | K6 | 1119-44-4 | 2E-09 | 1E-03 | 10× | ketone |
| 5-methyl-2-hepten-4-one | K7 | 81925-81-7 | 1E-09 | 1E-03 | 10× | ketone |
| 2-octanone | K8 | 111-13-7 | 1E-10 | 1E-04 | 10× | ketone |
| 3-octen-2-one | K9 | 1669-44-9 | 2E-10 | 1E-04 | 1× | ketone |
| 2-nonanone | K10 | 821-55-6 | 5E-10 | 1E-03 | 10× | ketone |
| 1-hexanol | L1 | 111-27-3 | 7E-10 | 1E-03 | 10× | alcohol |
| cis-3-hexenol | L2 | 928-96-1 | 8E-10 | 1E-03 | 10× | alcohol |
| geraniol | L3 | 106-24-1 | 1E-09 | 1E-02 | 10× | alcohol |
| R(-)1-octen-3-ol | L4 | 3687-48-7 | 4E-10 | 1E-03 | 10× | alcohol |
| benzaldehyde | AD1 | 100-52-7 | 8E-11 | 1E-04 | 10× | aromatic ald. |
| trans-cinnamaldehyde | AD2 | 14371-10-9 | 2E-11 | 1E-03 | 10× | aromatic ald. |
| cuminaldehyde | AD3 | 122-03-2 | 5E-11 | 1E-03 | 10× | aromatic ald. |
| p-anisaldehyde | AD4 | 123-11-5 | 8E-12 | 1E-05 | 10× | aromatic ald. |
| piperonal | AD5 | 120-57-0 | 2E-12 | 1E-03 | 1× | aromatic ald. |
| vanillin | AD6 | 121-33-5 | 3E-11 | 1E-01 | 10× | aromatic ald. |
| acetophenone | AK1 | 98-86-2 | 3E-12 | 1E-05 | 10× | aromatic ket./est. |
| 2'-hydroxyacetophenone | AK2 | 118-93-4 | 5E-12 | 1E-04 | 1× | aromatic ket./est. |
| 2,4-dimethylacetophenone | AK3 | 89-74-7 | 6E-13 | 1E-05 | 10× | aromatic ket./est. |
| 4-aminoacetophenone | AK4.M2 | 99-92-3 | 3E-10 | 1E+00 | 1× | mixed |
| 4-methylacetophenone | AK5 | 122-00-9 | 7E-12 | 1E-04 | 10× | aromaticket./est. |
| 4-methoxyacetophenone | AK6 | 100-06-1 | 5E-13 | 1E-04 | 1× | aromatic ket./est. |
| 2-methylacetophenone | AK7 | 577-16-2 | 1E-12 | 1E-05 | 10× | aromatic ket./est. |
| propiophenone | AK8 | 93-55-0 | 1E-11 | 1E-04 | 1× | aromatic ket./est. |
| butyrophenone | AK9 | 495-40-9 | 6E-12 | 1E-04 | 10× | aromatic ket./est. |
| 4-phenyl-2-butanone | AK10 | 2550-26-7 | 5E-11 | 1E-03 | 10× | aromatic ket./est. |
| 4-(4-hydroxyphenyl)-2-butanone | AK11 | 5471-51-2 | 8E-11 | 1E-01 | 10× | aromatic ket./est. |
| methyl salicylate | AE1 | 119-36-8 | 4E-11 | 1E-03 | 1× | aromatic ket./est. |
| methyl benzoate | AE2 | 93-58-3 | 3E-11 | 1E-04 | 10× | aromatic ket./est. |
| benzyl acetate | AE3 | 140-11-4 | 1E-10 | 1E-03 | 10× | aromatic ket./est. |
| ethyl benzoate | AE4 | 93-89-0 | 2E-11 | 1E-04 | 10× | aromatic ket./est. |
| benzyl benzoate | AE5 | 120-51-4 | 4E-11 | 1E-01 | 10× | aromatic ket./est. |
| methyl anthranilate | AE6.M3 | 134-20-3 | 1E-13 | 1E-05 | 10× | mixed |
| dimethyl anthranilate | AE7.M4 | 85-91-6 | 2E-11 | 1E-03 | 10× | mixed |
| phenyl acetate | AE8 | 122-79-2 | 3E-12 | 1E-05 | 10× | aromatic ket./est. |
| ethyl phenylacetate | AE9 | 101-97-3 | 7E-11 | 1E-03 | 1× | aromatic ket./est. |
| allyl phenylacetate | AE10 | 1797-74-6 | 2E-11 | 1E-03 | 10× | aromatic ket./est. |
| phenyl propionate | AE11 | 637-27-4 | 1E-11 | 1E-04 | 10× | aromatic ket./est. |
| m-cresol | AP1 | 108-39-4 | 1E-10 | 1E-03 | 1× | aromatic phen./moxy. |
| carvacrol | AP2 | 499-75-2 | 2E-09 | 1E-01 | 10× | aromatic phen./moxy. |
| 4-methylanisole | AP3 | 104-93-8 | 9E-11 | 1E-04 | 10× | aromatic phen./moxy. |
| guaiacol | AP4 | 90-05-1 | 9E-13 | 1E-05 | 1× | aromatic phen./moxy. |
| 2,6-dimethoxyphenol | AP5 | 91-10-1 | 2E-12 | 1E-03 | 1× | aromatic phen./moxy. |
| eugenol | AP6 | 97-53-0 | 7E-13 | 1E-04 | 10× | aromatic phen./moxy. |
| isoeugenol | AP7 | 97-54-1 | 8E-13 | 1E-04 | 10× | aromatic phen./moxy. |
| methyl eugenol | AP8 | 93-15-2 | 2E-12 | 1E-04 | 10× | aromatic phen./moxy. |
| methyl isoeugenol | AP9 | 93-16-3 | 8E-13 | 1E-04 | 10× | aromatic phen./moxy. |
| eugenyl acetate | AP10 | 93-28-7 | 4E-11 | 1E-02 | 10× | aromatic phen./moxy. |
| elemicin | AP11 | 487-11-6 | 5E-12 | 1E-03 | 10× | aromatic phen./moxy. |
| fenchol | CT1 | 1632-73-1 | 5E-10 | 1E-02 | 1× | cyclic terpenoid |
| 2-ethyl fenchol | CT2 | 18368-91-7 | 6E-11 | 1E-02 | 10× | cyclic terpenoid |
| alpha-pinene | CT3 | 7785-70-8 | 3E-09 | 1E-03 | 10× | cyclic terpenoid |
| isobornyl isovalerate | CT4.M5 | 7779-73-9 | 1E-09 | 1E-01 | 10× | mixed |
| 1,8-cineole | CT5 | 470-82-6 | 1E-09 | 1E-03 | 10× | cyclic terpenoid |
| L-carvone | CT6 | 6485-40-1 | 1E-10 | 1E-03 | 10× | cyclic terpenoid |
| beta-ionone | CT7 | 14901-07-6 | 1E-11 | 1E-03 | 10× | cyclic terpenoid |
| beta-damascone | CT8 | 23726-91-2 | 1E-11 | 1E-03 | 10× | cyclic terpenoid |
| damascenone | CT9 | 23696-85-7 | 2E-10 | 1E-02 | 10× | cyclic terpenoid |
| menthone | CT10 | 10458-14-7 | 3E-10 | 1E-03 | 10× | cyclic terpenoid |
| (R)-(+)-pulegone | CT11 | 89-82-7 | 7E-13 | 1E-05 | 10× | cyclic terpenoid |
| (+)-isomenthone | CT12 | 1196-31-2 | 2E-10 | 1E-03 | 10× | cyclic terpenoid |
| nootkatone | CT13 | 4674-50-4 | 9E-10 | 1E+00 | 1× | cyclic terpenoid |
| (+-)-menthol | CT14 | 89-78-1 | 6E-11 | 1E-02 | 10× | cyclic terpenoid |
| (+)-neomenthol | CT15 | 2216-52-6 | 2E-10 | 1E-02 | 10× | cyclic terpenoid |
| (+-)-geosmin | CT16 | 16423-19-1 | 4E-11 | 1E-01 | 10× | cyclic terpenoid |
| (-)-ambroxide | CT17 | 6790-58-5 | 7E-10 | 1E-01 | 10× | cyclic terpenoid |
| (+)-menthofuran | CT18.M6 | 17957-94-7 | 2E-10 | 1E-03 | 10× | mixed |
| 2-pentylfuran | F1 | 3777-69-3 | 2E-09 | 1E-03 | 10× | furan/pyrone/lactone |
| 5-ethyl-4-hydroxy-2-methyl-3(2H)-furanone | F2 | 27538-09-6 | 9E-13 | 1E-04 | 10× | furan/pyrone/lactone |
| 5-methylfurfural | F3 | 620-02-0 | 5E-10 | 1E-03 | 10× | furan/pyrone/lactone |
| ethyl maltol | F4 | 4940-11-8 | 1E-12 | 1E-02 | 1× | furan/pyrone/lactone |
| gamma-undecalactone | F5 | 104-67-6 | 3E-09 | 1E+00 | 1× | furan/pyrone/lactone |
| coumarin | F6 | 91-64-5 | 7E-10 | 1E-02 | 10× | furan/pyrone/lactone |
| 1-furfurylpyrrole | F7.M7 | 1438-94-4 | 1E-10 | 1E-02 | 10× | mixed |
| ethylenediamine | N1 | 107-15-3 | 1E-10 | 1E-05 | 10× | amine |
| butylamine | N2 | 109-73-9 | 6E-10 | 1E-05 | 10× | amine |
| 2-methylbutylamine | N3 | 96-15-1 | 4E-10 | 1E-05 | 10× | amine |
| N-butyldimethylamine | N4 | 927-62-8 | 3E-10 | 1E-05 | 10× | amine |
| isopentylamine | N5 | 107-85-7 | 3E-11 | 1E-06 | 10× | amine |
| cadaverine | N6 | 462-94-2 | 7E-13 | 1E-06 | 10× | amine |
| octylamine | N7 | 111-86-4 | 7E-11 | 1E-04 | 10× | amine |
| N,N-dimethyloctylamine | N8 | 7378-99-6 | 6E-12 | 1E-05 | 10× | amine |
| cyclohexylamine | N9 | 108-91-8 | 6E-11 | 1E-05 | 10× | amine |
| N,N-dimethylcyclohexylamine | N10 | 98-94-2 | 2E-11 | 1E-05 | 10× | amine |
| β-phenylethylamine | N11 | 64-04-0 | 3E-13 | 1E-06 | 1× | amine |
| benzylamine | N12 | 100-46-9 | 5E-11 | 1E-04 | 10× | amine |
| *tyramine* | *N13* | *51-67-2* | *2E-09* | *1E+00* | 1× | *amine* |
| N,N-dimethyl-2-phenethylamine | N14 | 1126-71-2 | 2E-13 | 1E-06 | 10× | amine |
| 3-phenylpropylamine | N15 | 2038-57-5 | 1E-11 | 1E-04 | 10× | amine |
| N-methyl piperidine | N16 | 626-67-5 | 2E-10 | 1E-05 | 1× | amine |
| 5-methyl heptan-3-one oxime | N17.M8 | 22457-23-4 | 3E-10 | 1E-02 | 10× | mixed |
| pyrazine | P1 | 290-37-9 | 2E-09 | 1E-04 | 10× | pyrazine |
| 2-acetylpyrazine | P2 | 22047-25-2 | 1E-09 | 1E-02 | 10× | pyrazine |
| 2-methylpyrazine | P3 | 109-08-0 | 7E-11 | 1E-05 | 10× | pyrazine |
| 2-methoxypyrazine | P4 | 3149-28-8 | 3E-11 | 1E-05 | 10× | pyrazine |
| 2-ethylpyrazine | P5 | 13925-00-3 | 3E-11 | 1E-05 | 10× | pyrazine |
| 2,3-diethylpyrazine | P6 | 15707-24-1 | 6E-10 | 1E-03 | 1× | pyrazine |
| 2-chloropyrazine | P7 | 14508-49-7 | 2E-09 | 1E-03 | 10× | pyrazine |
| pyrazineethanethiol | P8 | 35250-53-4 | 4E-11 | 1E-03 | 10× | pyrazine |
| 2-methoxy-3-methylpyrazine | P9 | 2847-30-5 | 5E-11 | 1E-04 | 10× | pyrazine |
| 2-ethyl-3-methylpyrazine | P10 | 15707-23-0 | 5E-11 | 1E-04 | 10× | pyrazine |
| 2,3-dimethylpyrazine | P11 | 5910-89-4 | 3E-10 | 1E-04 | 10× | pyrazine |
| 2,5-dimethylpyrazine | P12 | 123-32-0 | 3E-10 | 1E-04 | 10× | pyrazine |
| 2,6-dimethylpyrazine | P13 | 108-50-9 | 3E-09 | 1E-03 | 10× | pyrazine |
| 2,3,5-trimethylpyrazine | P14 | 14667-55-1 | 1E-11 | 1E-05 | 10× | pyrazine |
| 2,3,5,6-tetramethylpyrazine | P15 | 1124-11-4 | 5E-10 | 1E-03 | 10× | pyrazine |
| 2-ethyl-5-methylpyrazine | P16 | 13360-64-0 | 2E-11 | 1E-05 | 10× | pyrazine |
| 2-acetyl-3,(5 or 6)-dimethylpyrazine | P17 | 54300-08-2 | 2E-09 | 1E-03 | 1× | pyrazine |
| 2-isobutyl-3-methylpyrazine | P18 | 13925-06-9 | 2E-10 | 1E-03 | 10× | pyrazine |
| 2-acetyl-3-ethylpyrazine | P19 | 32974-92-8 | 2E-10 | 1E-02 | 1× | pyrazine |
| 2-acetyl-3-methylpyrazine | P20 | 23787-80-6 | 8E-11 | 1E-03 | 10× | pyrazine |
| 2-ethyl-3-methoxypyrazine | P21 | 25680-58-4 | 1E-11 | 1E-05 | 10× | pyrazine |
| 2-methoxy-3(5 or 6)-isopropylpyrazine | P22 | 93905-03-4 | 3E-11 | 1E-04 | 10× | pyrazine |
| 2-isobutyl-3-methoxypyrazine | P23 | 24683-00-9 | 3E-11 | 1E-03 | 10× | pyrazine |
| 2-methoxy-3-(1-methylpropyl)pyrazine | P24 | 24168-70-5 | 3E-11 | 1E-03 | 10× | pyrazine |
| 2-isopropyl-3-methoxypyrazine | P25 | 25773-40-4 | 9E-12 | 1E-04 | 10× | pyrazine |
| 5H-5-methyl-6,7-dihydrocyclopenta[b]pyrazine | P26 | 23747-48-0 | 2E-10 | 1E-03 | 1× | pyrazine |
| 5,6,7,8-tetrahydroquinoxaline | P27 | 34413-35-9 | 4E-11 | 1E-03 | 10× | pyrazine |
| 5-methylquinoxaline | P28 | 13708-12-8 | 4E-10 | 1E-02 | 10× | pyrazine |
| 2-acetylpyridine | NS1 | 1122-62-9 | 4E-11 | 1E-04 | 1× | heterocyclic N-S |
| 4-tert-butylpyridine | NS2 | 3978-81-2 | 4E-12 | 1E-05 | 10× | heterocyclic N-S |
| indole | NS3 | 120-72-9 | 9E-11 | 1E-02 | 10× | heterocyclic N-S |
| 3-methylindole | NS4 | 83-34-1 | 2E-11 | 1E-02 | 10× | heterocyclic N-S |
| 2-isobutylthiazole | NS5 | 18640-74-9 | 3E-12 | 1E-05 | 10× | heterocyclic N-S |
| 2-acetylthiazole | NS6 | 24295-03-2 | 1E-10 | 1E-03 | 10× | heterocyclic N-S |
| 2-isopropyl-4-methylthiazole | NS7 | 15679-13-7 | 3E-12 | 1E-05 | 10× | heterocyclic N-S |
| 2-methyl-2-thiazoline | NS8 | 2346-00-1 | 4E-10 | 1E-04 | 10× | heterocyclic N-S |
| 2,4,5-trimethylthiazole | NS9 | 13623-11-5 | 5E-12 | 1E-05 | 10× | heterocyclic N-S |
| ethyl-2,5-dihydro-4-methylthiazole | NS10 | 41803-21-8 | 6E-11 | 1E-04 | 10× | heterocyclic N-S |
| 4-methylthiazole | NS11 | 693-95-8 | 8E-10 | 1E-04 | 10× | heterocyclic N-S |
| 2-methyl-4-propyl-1,3-oxathiane | NS12.M9 | 67715-80-4 | 9E-11 | 1E-03 | 1× | mixed |
| dimethyl trisulfide | S1 | 3658-80-8 | 8E-10 | 1E-03 | 10× | sulfide-thiol |
| *allyl disulfide* | *S2* | *2179-57-9* | *7E-10* | *1E-03* | *10×* | *sulfide-thiol* |
| 4-methoxy-2-methyl-2-butanethiol | S3 | 94087-83-9 | 3E-12 | 1E-06 | 10× | sulfide-thiol |
| methional | S4.M10 | 3268-49-3 | 1E-11 | 1E-05 | 10× | mixed |
| 3-mercaptohexyl acetate | S5 | 136954-20-6 | 4E-12 | 1E-04 | 10× | sulfide-thiol |
| 3-(methylthio)-1-hexanol | S6 | 51755-66-9 | 6E-09 | 1E-02 | 10× | sulfide-thiol |
| furfuryl mercaptan | S7.M11 | 98-02-2 | 3E-12 | 1E-06 | 10× | mixed |
| difurfuryl disulfide | S8.M12 | 4437-20-1 | 4E-13 | 1E-03 | 10× | mixed |
| furfuryl methyl sulfide | S9.M13 | 1438-91-1 | 1E-09 | 1E-03 | 10× | mixed |
| 2-methyl-3-tetrahydrofuranthiol | S10 | 57124-87-5 | 2E-10 | 1E-04 | 10× | sulfide-thiol |
| myrcene | ENE1 | 123-35-3 | 2E-09 | 1E-03 | 10× | alkene |
| 1,3,5-undecatriene | ENE2 | 16356-11-9 | 5E-09 | 1E-02 | 10× | alkene |
| (R)-(+)-limonene | ENE3 | 5989-27-5 | 1E-09 | 1E-03 | 10× | alkene |
| empty | - | - | - | - | - | control |
| triglyceride | - | - | - | - | - | control |

**Supplementary File 1. Table of odorants and estimated concentrations used in the study.**

**Code:** abbreviated identifier code, used in Fig. 5A and Appendix 1– Figure 1. **Epifl. est. conc.:** estimated delivered concentration of odorant vapor, in mols/L (M), for 1x dataset. Most commonly-presented values (of 4 preparations) are shown, reported to one significant digit precision. **Dilution:** liquid dilution of odorant used to generate delivered concentration. **Two-photon rel. conc.:** concentration used for the two-photon imaging dataset relative to the concentration used in the widefield epifluorescence dataset. **Class:** nominal odorant classification based on structural features. Odorants in italics gave no response at the given concentration in any of the 8 OBs.
