# Supplementary material for: Mapping odorant sensitivities reveals a sparse but structured representation of olfactory chemical space by sensory input to the mouse olfactory bulb
Source: eLife. 2022 Jul 21;11:e80470. doi: 10.7554/eLife.80470 (PMC9352350; doi:10.7554/eLife.80470)
Supplement: Supplementary file 2. — Compilation of olfactory sensory neuron response maps to the 185-odorant panel imaged in each of four OMP-IRES-tTA; tetO-GCaMP6s mice. See Materials and methods for details of odorant presentation, imaging and map generation. Each map is scaled to its own maximum (mean of highest 65 pixels) and clipped at ΔF=0. Concentrations are given as estimated delivered concentration to the mouse nose, rounded to the nearest order of magnitude to allow for deviations from ideal behavior of the odorant in its solvent. See Supplementary file 1 for more precise estimates of delivered concentration. Grid overlay is for facilitating visual comparison across maps. Odorants are grouped by major structural features and are numbered in order of presentation in Main figures and Supplementary file 1. Letter-number identifier matches that in Figure 5A and in Supplementary file 1. Maps with no measured response in any glomerulus of a given mouse are shown at 50% opacity. Odorants failing to evoke a response in all four mice are indicated with text. [file elife-80470-supp2.pdf]

Atlas of odorant response maps (185 odorants)

Carboxylic acid

MOUSE 1 | MOUSE 2 | MOUSE 3 | MOUSE 4

1. Propionic acid (C1)

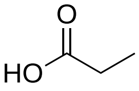

10 pM | 10 pM | 10 pM | 10 pM

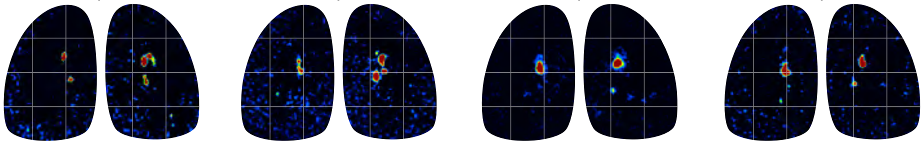

2. Butyric acid (C2)

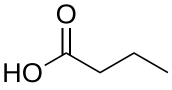

10 pM | 10 pM | 10 pM | 10 pM

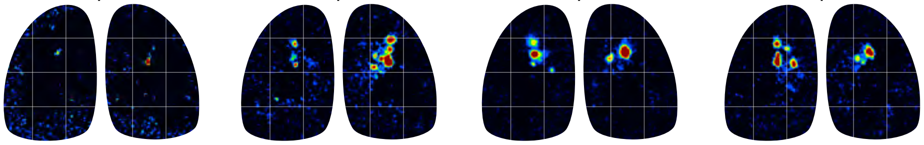

3. 2-methylbutyric acid (C3)

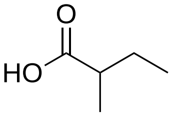

1 pM | 1 pM | 1 pM | 1 pM

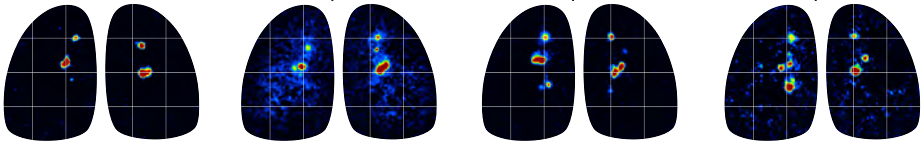

4. Valeric acid (C4)

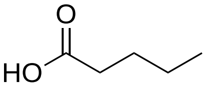

1 pM | 1 pM | 1 pM | 1 pM

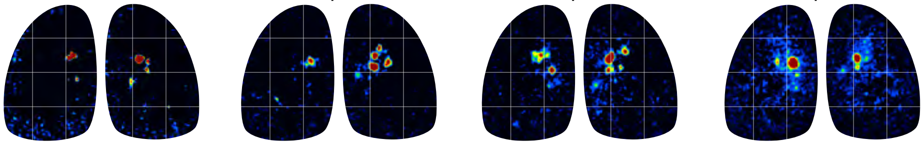

5. Isovaleric acid (C5)

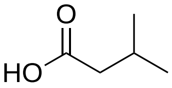

10 pM | 10 pM | 10 pM | 10 pM

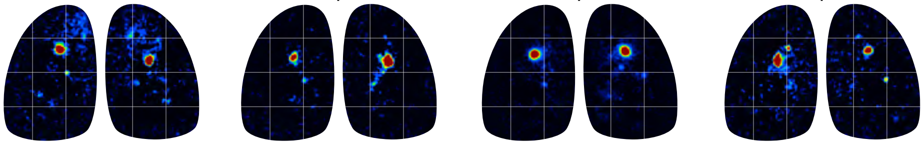

6. 2-methyl-2-pentenoic acid (C6)

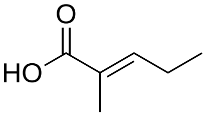

0.01 pM | 0.01 pM | 0.01 pM | 0.01 pM

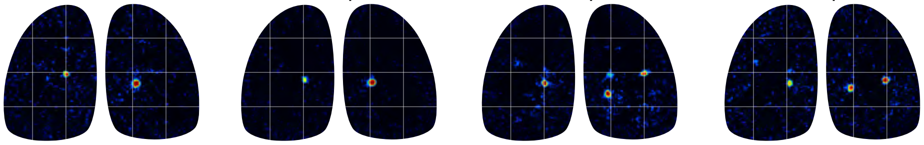

7. Hexanoic acid (C7)

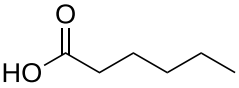

10 pM | 10 pM | 10 pM | 10 pM

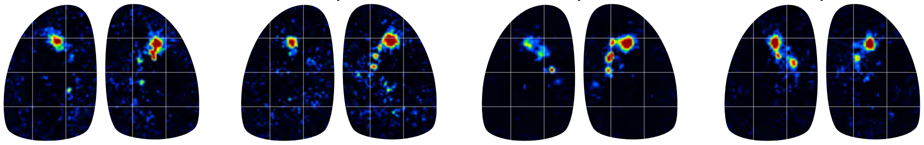

8. Heptanoic acid (C8)

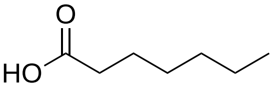

100 pM | 100 pM | 100 pM | 100 pM

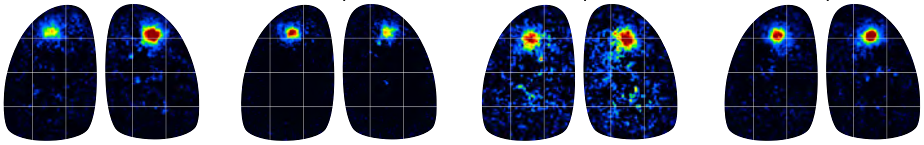

Aldehyde

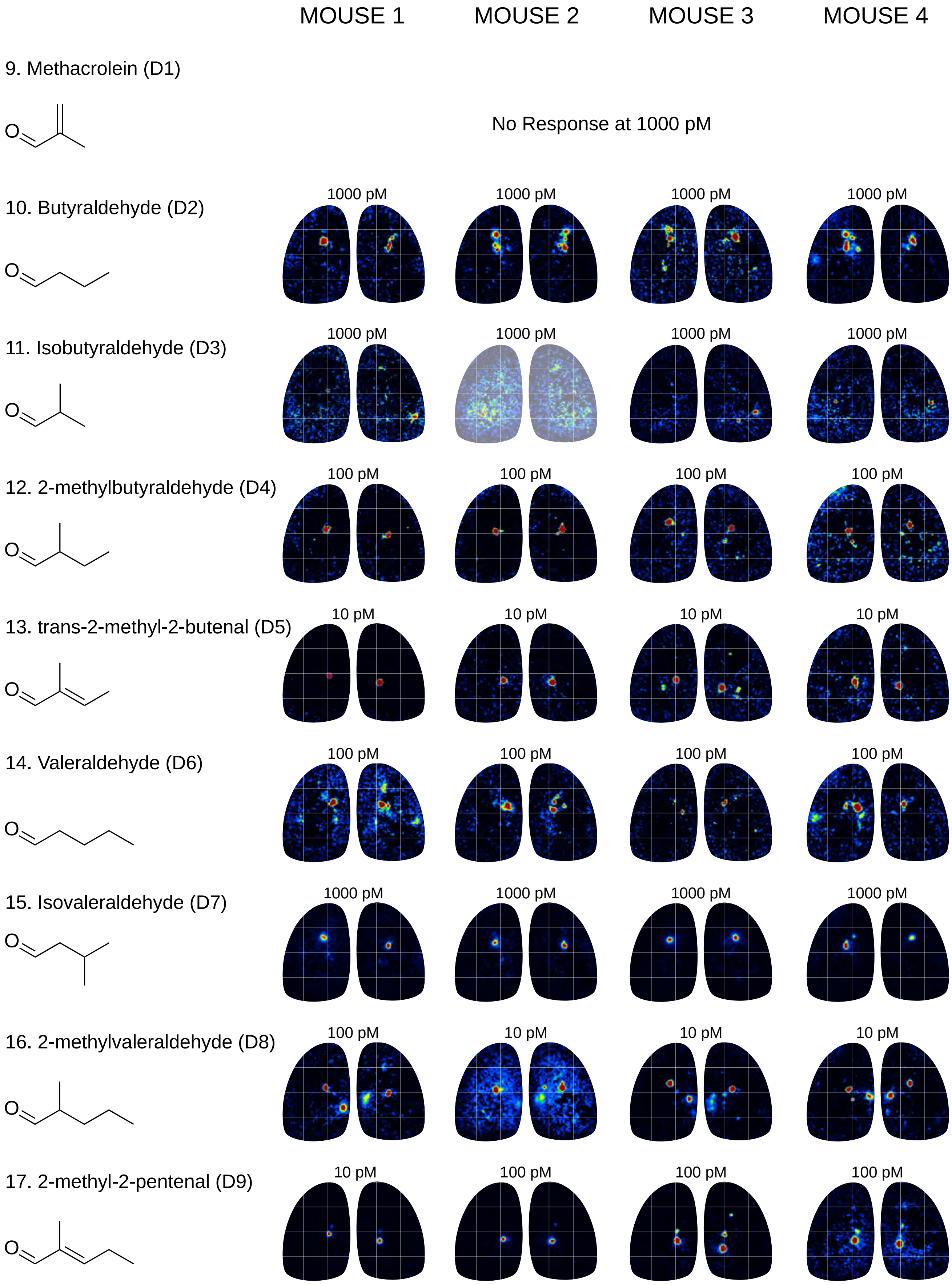
MOUSE 1
MOUSE 2
MOUSE 3
MOUSE 4
9. Methacrolein (D1)
No Response at 1000 pM
10. Butyraldehyde (D2)
1000 pM
1000 pM
1000 pM
1000 pM
11. Isobutyraldehyde (D3)
1000 pM
1000 pM
1000 pM
1000 pM
12. 2-methylbutyraldehyde (D4)
100 pM
100 pM
100 pM
100 pM
13. trans-2-methyl-2-butenal (D5)
10 pM
10 pM
10 pM
10 pM
14. Valeraldehyde (D6)
100 pM
100 pM
100 pM
100 pM
15. Isovaleraldehyde (D7)
1000 pM
1000 pM
1000 pM
1000 pM
16. 2-methylvaleraldehyde (D8)
100 pM
10 pM
10 pM
10 pM
17. 2-methyl-2-pentenal (D9)
10 pM
100 pM
100 pM
100 pM
O

Aldehyde II

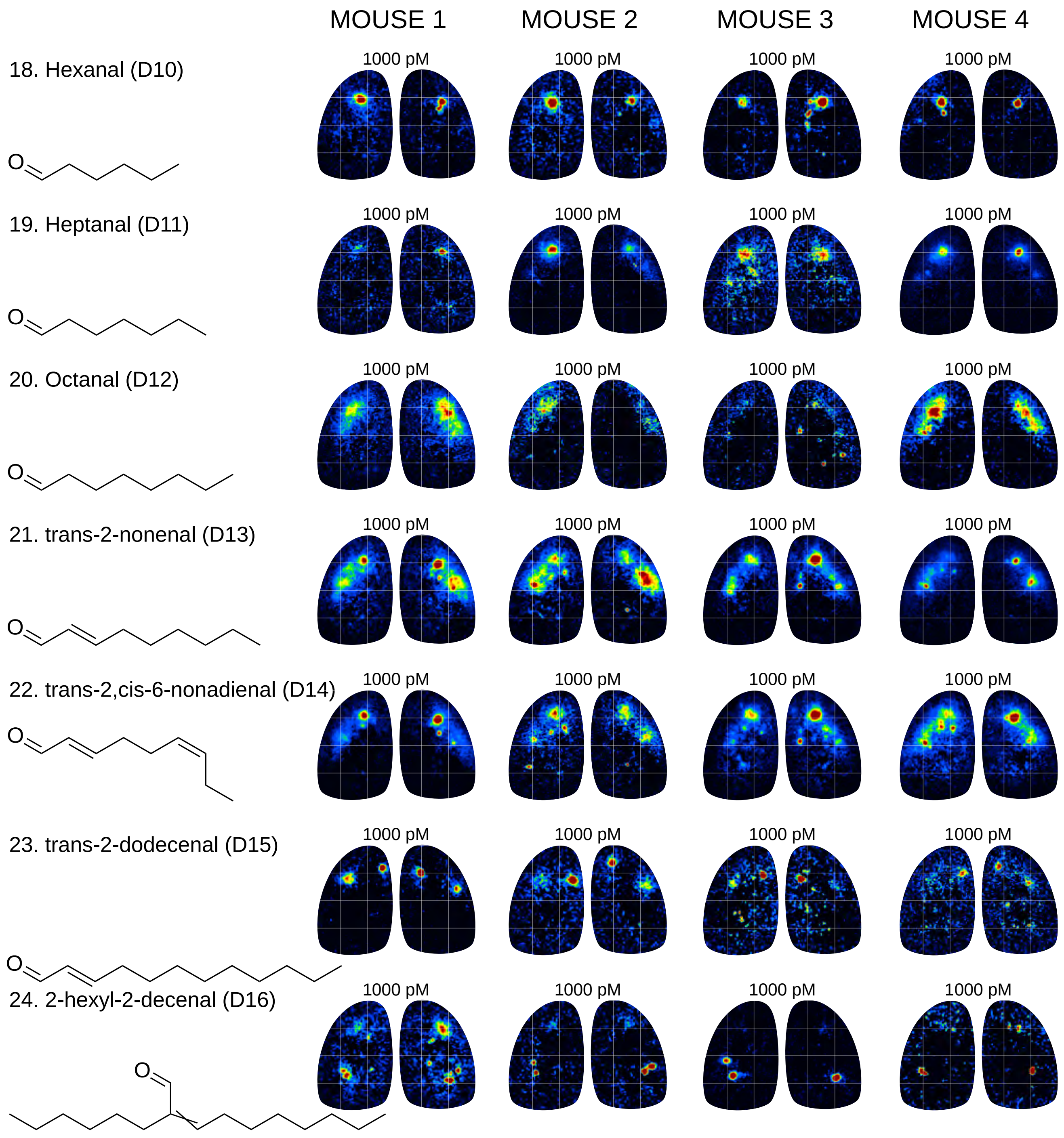
MOUSE 1
MOUSE 2
MOUSE 3
MOUSE 4
18. Hexanal (D10)
1000 pM
1000 pM
1000 pM
1000 pM
19. Heptanal (D11)
1000 pM
1000 pM
1000 pM
1000 pM
20. Octanal (D12)
1000 pM
1000 pM
1000 pM
1000 pM
21. trans-2-nonenal (D13)
1000 pM
1000 pM
1000 pM
1000 pM
22. trans-2,cis-6-nonadienal (D14)
1000 pM
1000 pM
1000 pM
1000 pM
23. trans-2-dodecenal (D15)
1000 pM
1000 pM
1000 pM
1000 pM
24. 2-hexyl-2-decenal (D16)
1000 pM
1000 pM
1000 pM
1000 pM

Ester

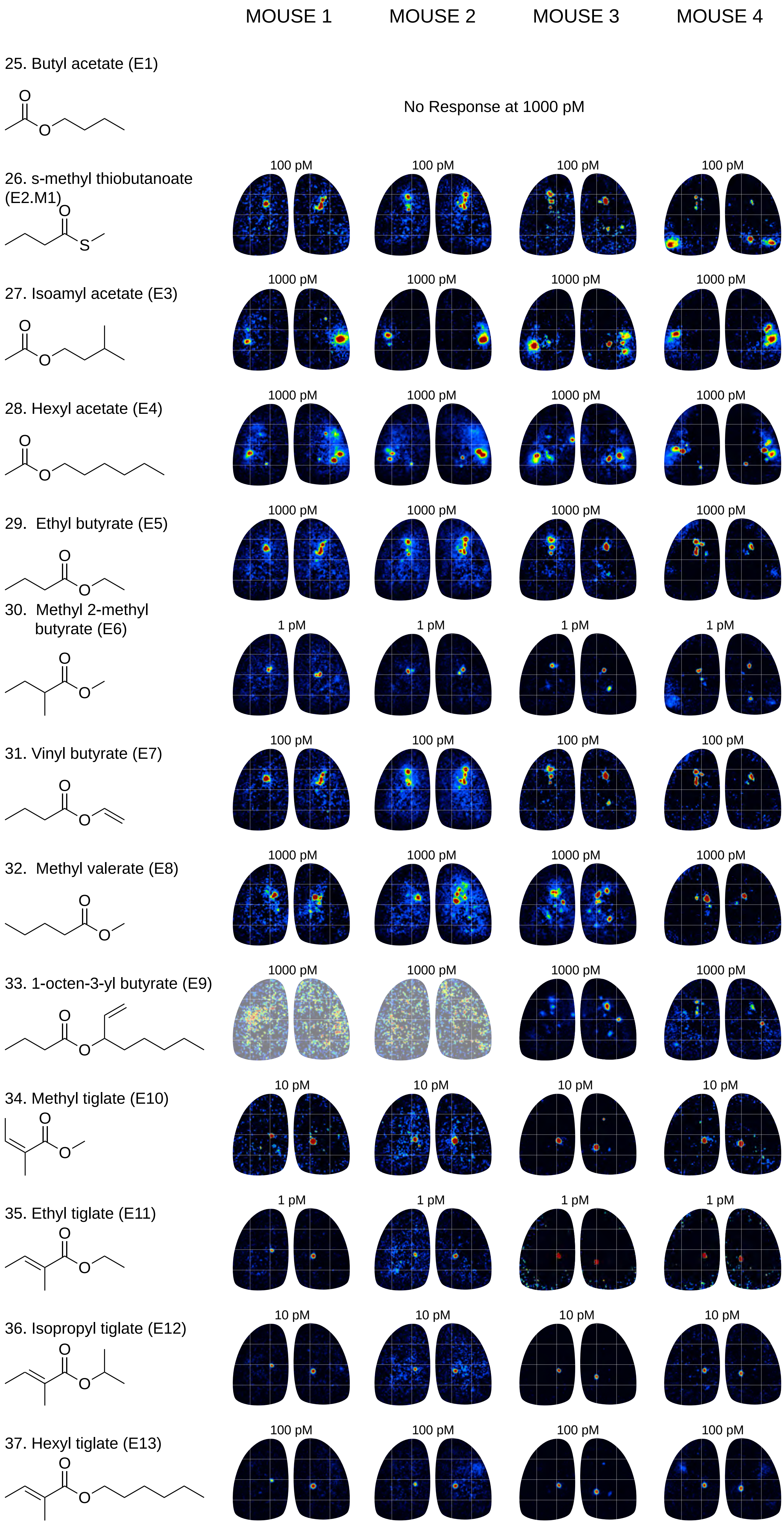

MOUSE 1
MOUSE 2
MOUSE 3
MOUSE 4
25. Butyl acetate (E1)
No Response at 1000 pM
26. s-methyl thiobutanoate (E2.M1)
100 pM
27. Isoamyl acetate (E3)
1000 pM
28. Hexyl acetate (E4)
1000 pM
29. Ethyl butyrate (E5)
1000 pM
30. Methyl 2-methyl butyrate (E6)
1 pM
31. Vinyl butyrate (E7)
100 pM
32. Methyl valerate (E8)
1000 pM
33. 1-octen-3-yl butyrate (E9)
1000 pM
34. Methyl tiglate (E10)
10 pM
35. Ethyl tiglate (E11)
1 pM
36. Isopropyl tiglate (E12)
10 pM
37. Hexyl tiglate (E13)
100 pM

Ketone

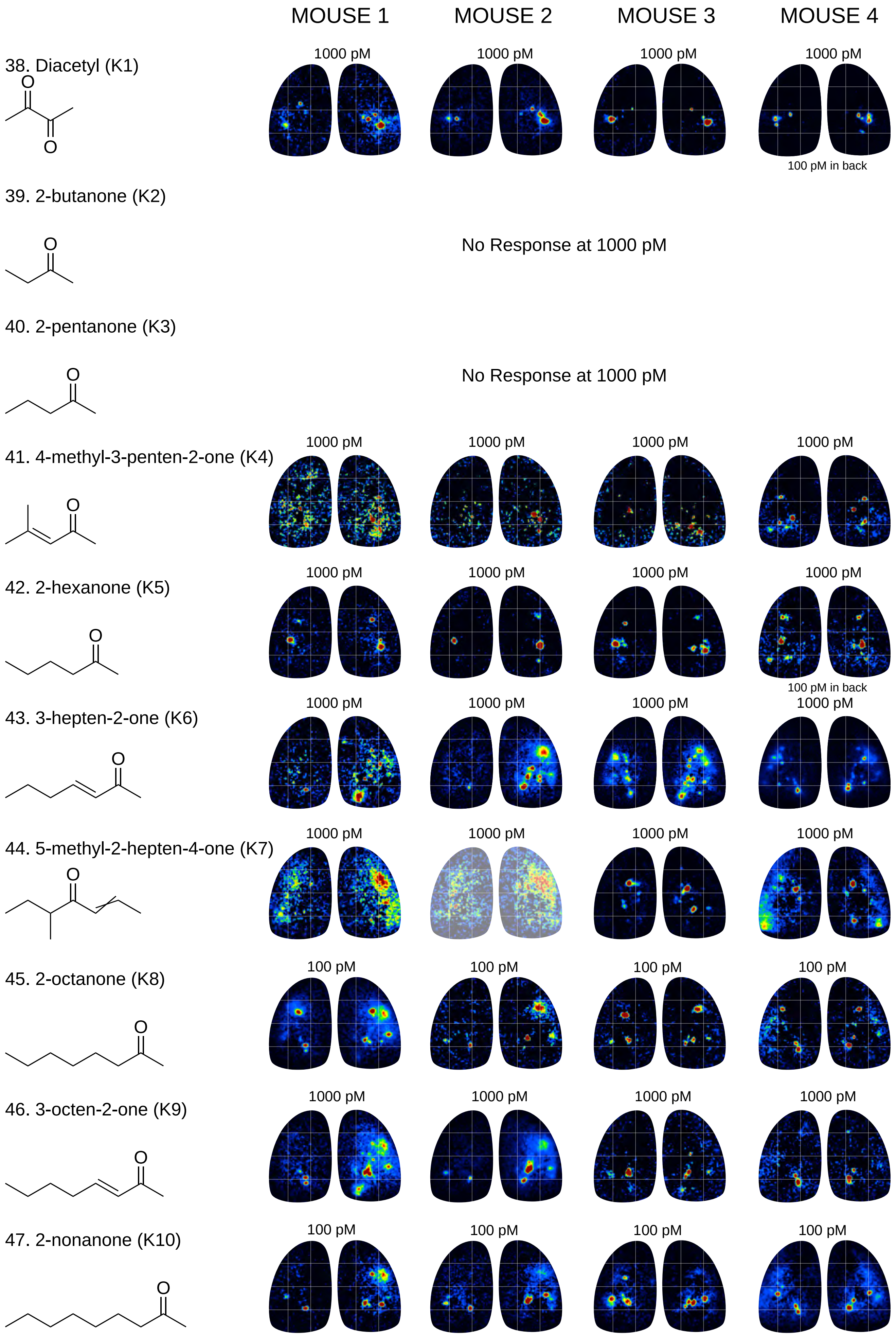

MOUSE 1
MOUSE 2
MOUSE 3
MOUSE 4
38. Diacetyl (K1)
1000 pM
1000 pM
1000 pM
1000 pM
100 pM in back
39. 2-butanone (K2)
No Response at 1000 pM
40. 2-pentanone (K3)
No Response at 1000 pM
41. 4-methyl-3-penten-2-one (K4)
1000 pM
1000 pM
1000 pM
1000 pM
42. 2-hexanone (K5)
1000 pM
1000 pM
1000 pM
1000 pM
100 pM in back
43. 3-hepten-2-one (K6)
1000 pM
1000 pM
1000 pM
1000 pM
44. 5-methyl-2-hepten-4-one (K7)
1000 pM
1000 pM
1000 pM
1000 pM
45. 2-octanone (K8)
100 pM
100 pM
100 pM
100 pM
46. 3-octen-2-one (K9)
1000 pM
1000 pM
1000 pM
1000 pM
47. 2-nonanone (K10)
100 pM
100 pM
100 pM
100 pM

Alcohol

MOUSE 1 MOUSE 2 MOUSE 3 MOUSE 4

48.1-hexanol (L1)

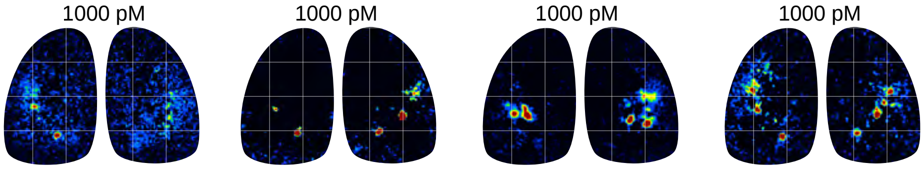

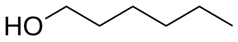

49. cis-3-hexanol (L2)

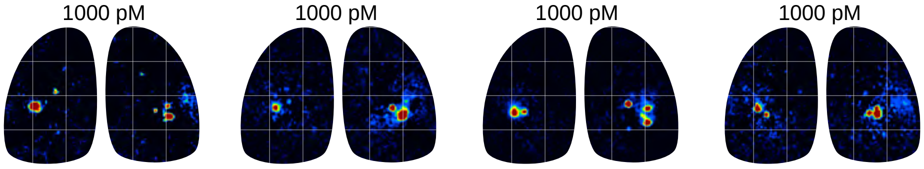

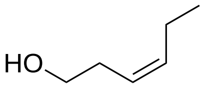

50. Geraniol (L3)

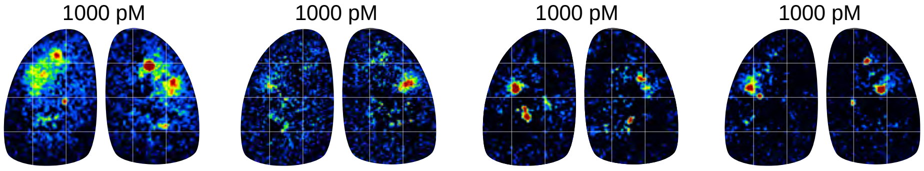

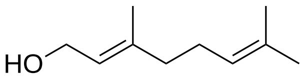

51. R(-)1-octen-3-ol (L4)

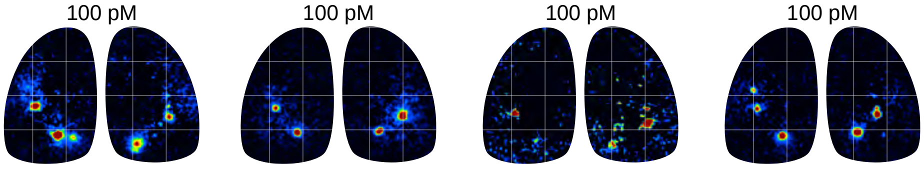

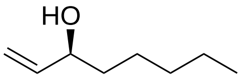

Aromatic Aldehyde

52.Benzaldehyde (AD1)

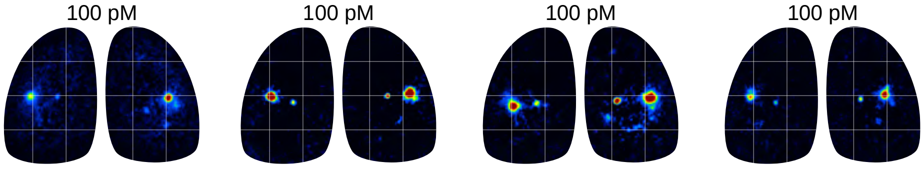

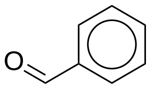

53. trans-cinnamaldehyde (AD2)

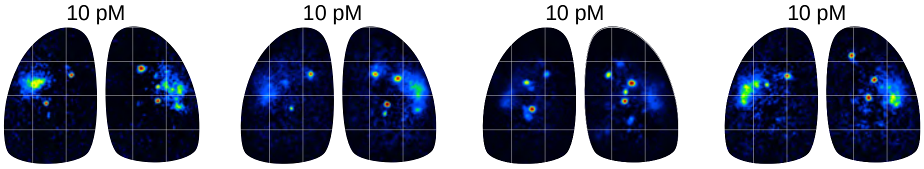

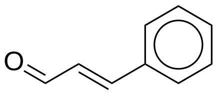

54. Cuminaldehyde (AD3)

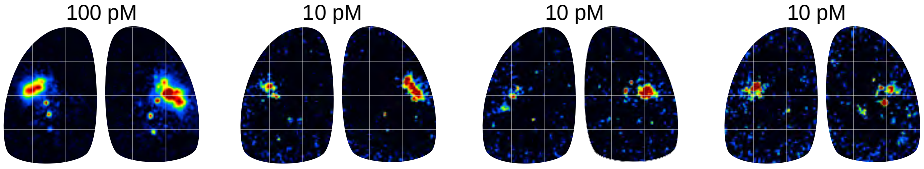

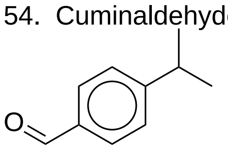

55. p-anisaldehyde (AD4)

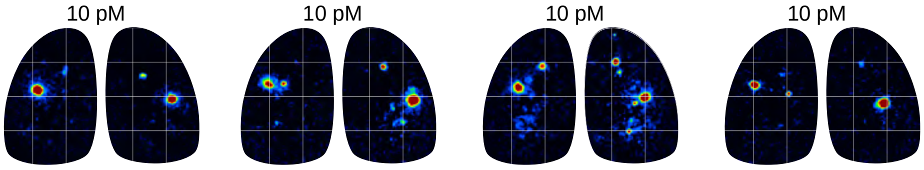

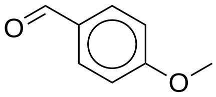

56. Piperonal (AD5)

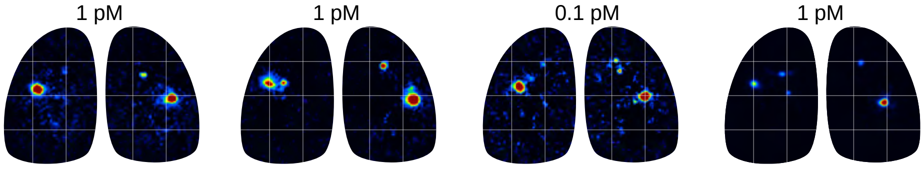

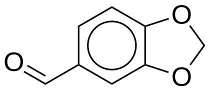

57. Vanillin (AD6)

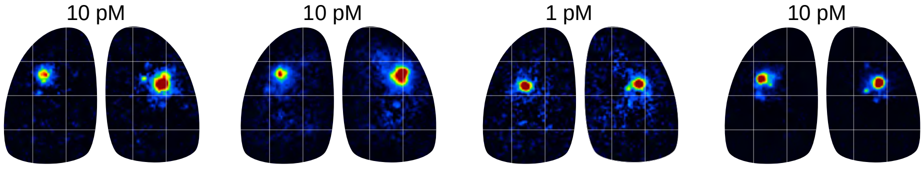

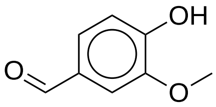

Aromatic Ketone

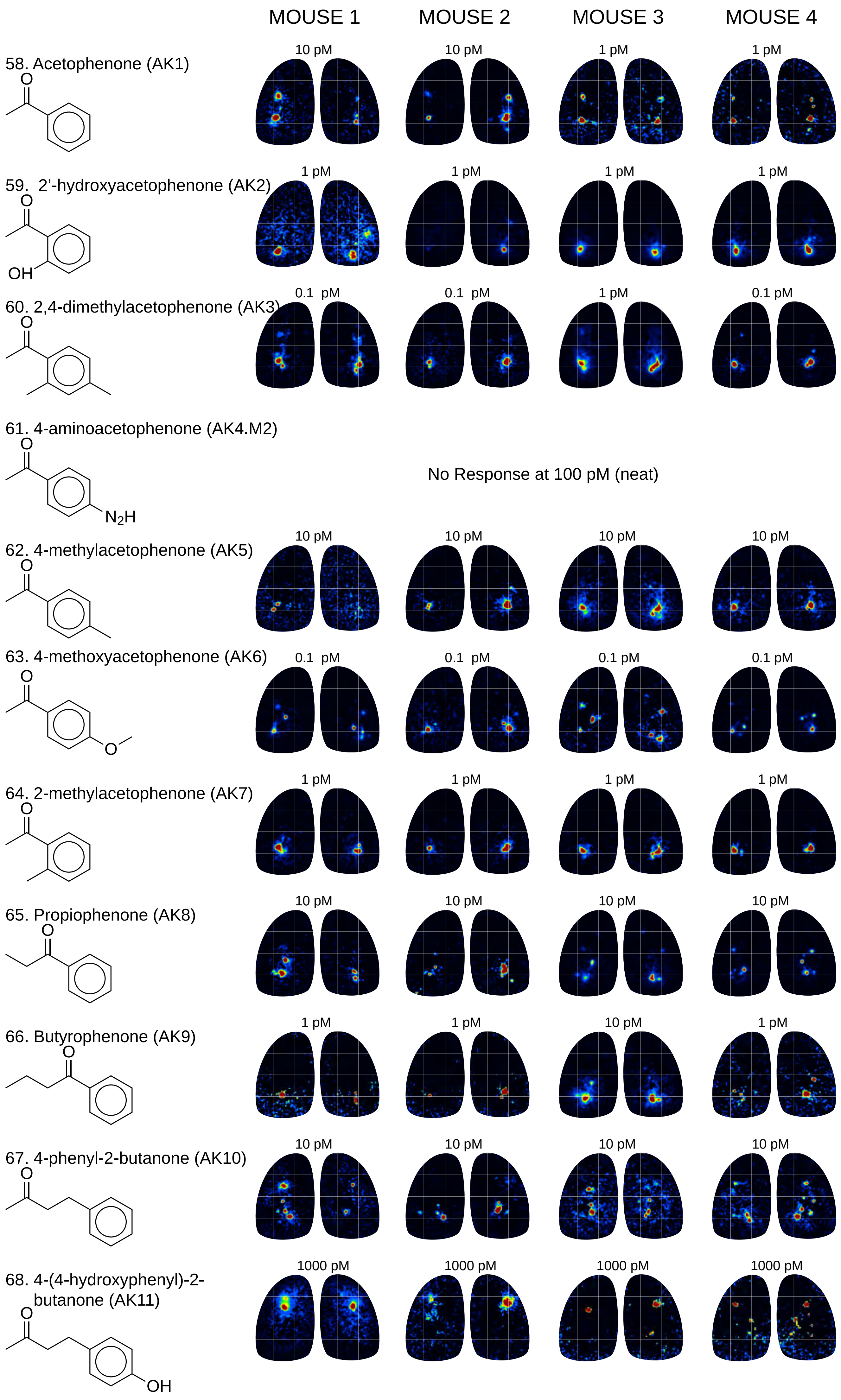

Aromatic Ester

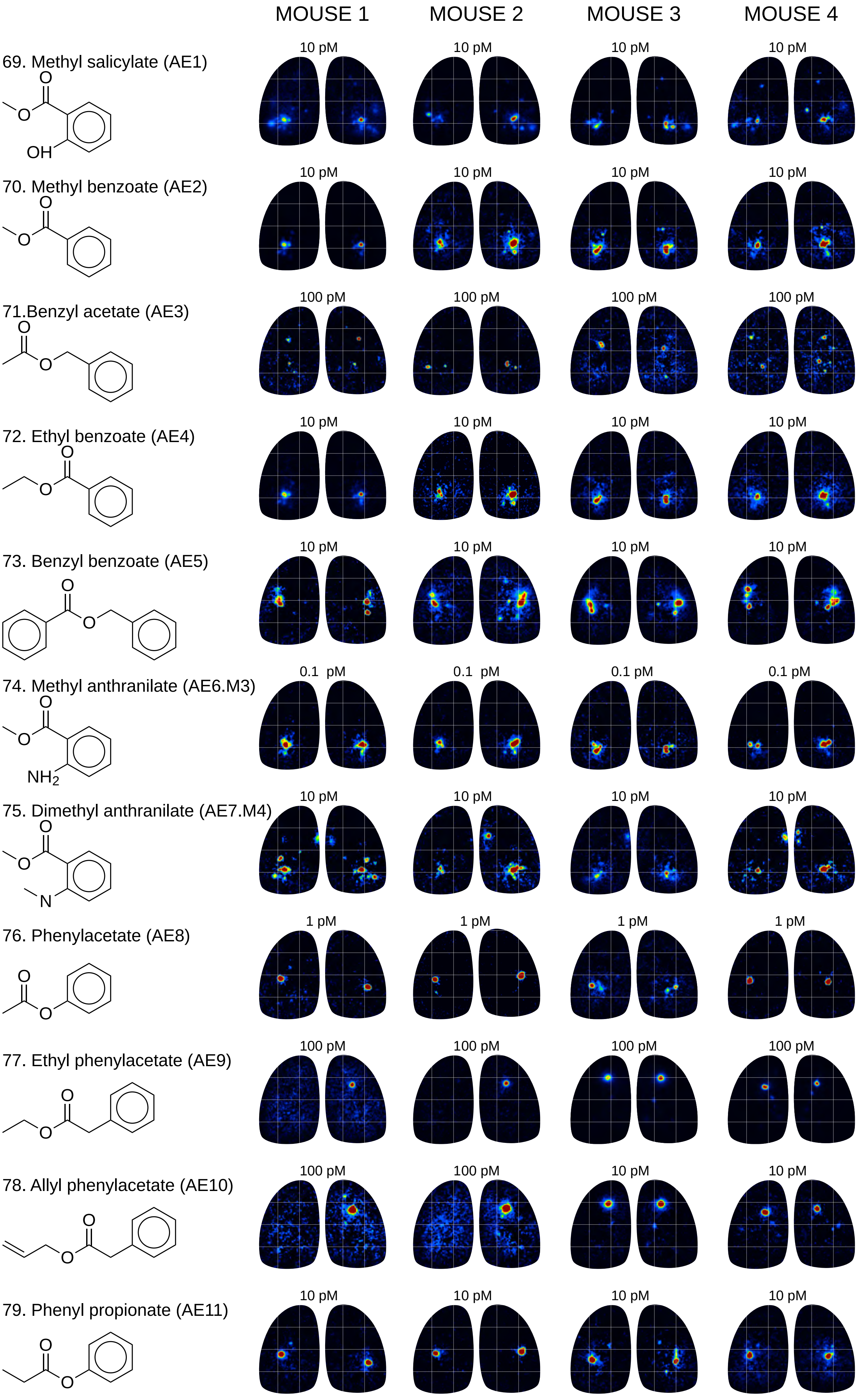

Aromatic Phenol/Methoxy

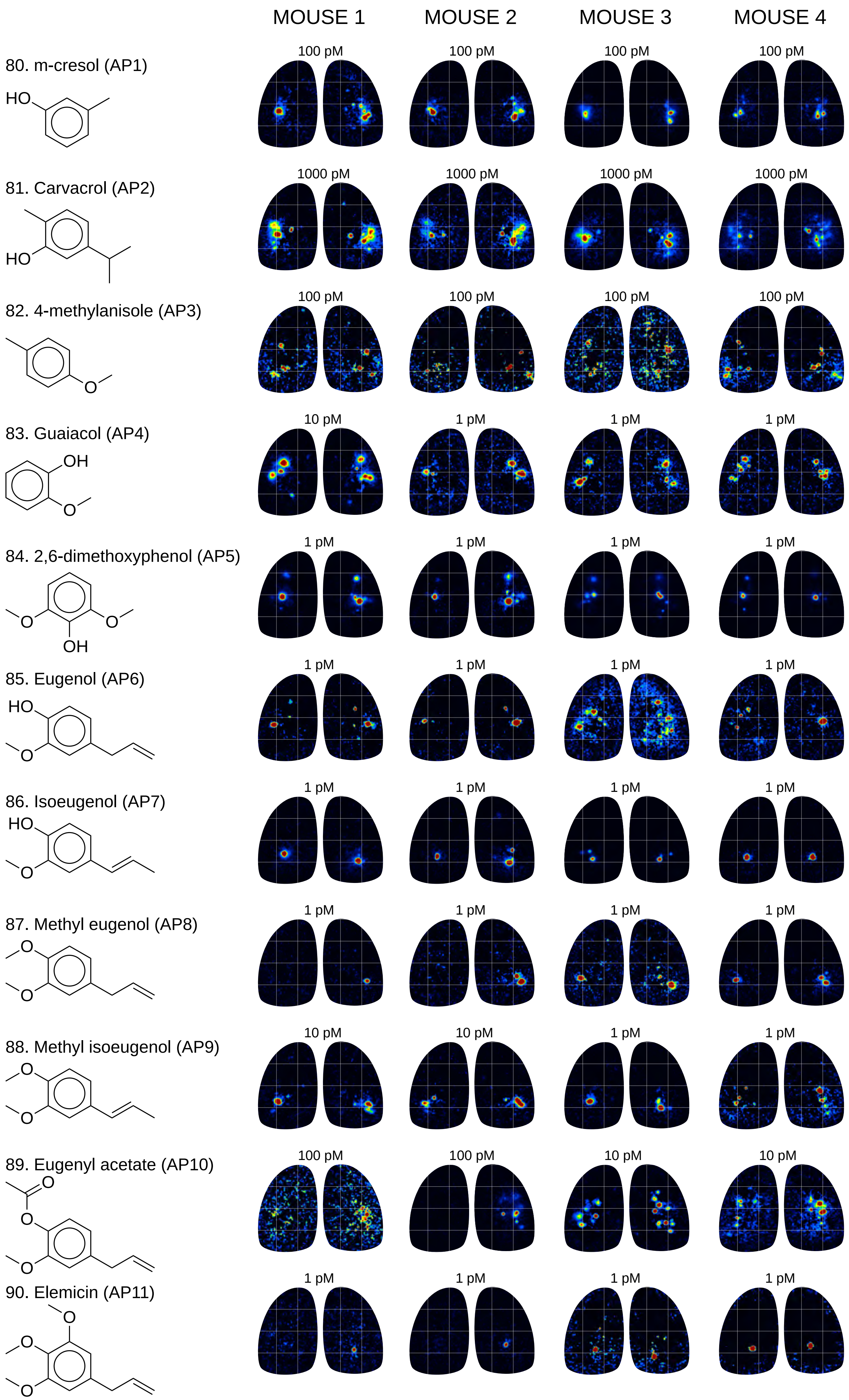

Cyclic Terpenoid I

	MOUSE 1	MOUSE 2	MOUSE 3	MOUSE 4

91. Fenchol (CT1)

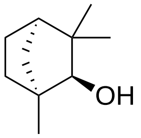

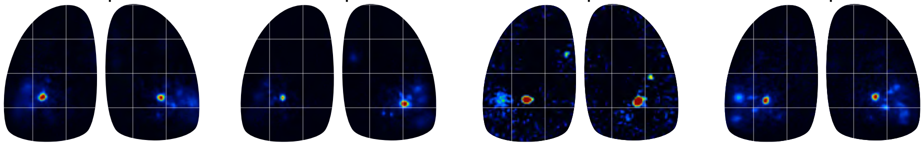

92. 2-ethyl fenchol (CT2)

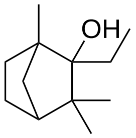

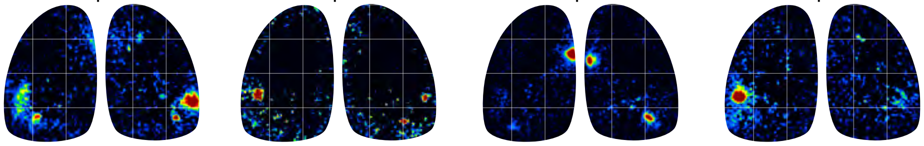

93. α-pinene (CT3)

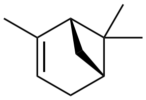

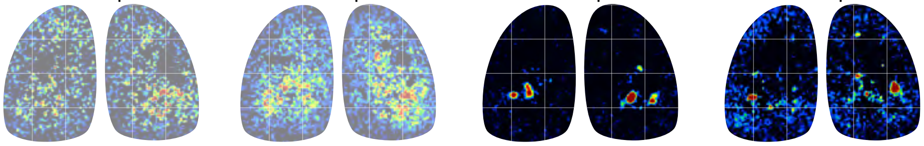

94. Isobornyl isovalerate (CT4.M5)

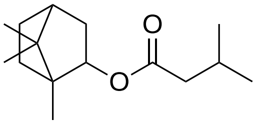

No Response at 1000 pM

95. 1,8-cineole (CT5)

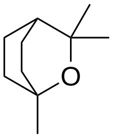

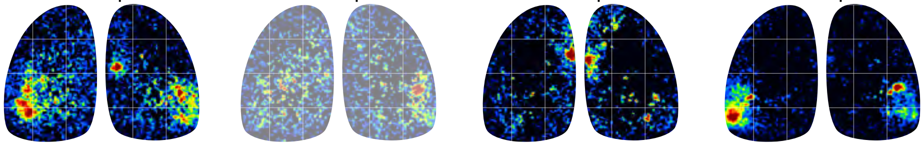

96. L-carvone (CT6)

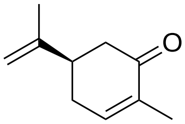

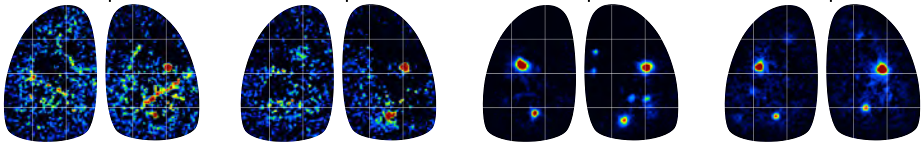

97. β-Ionone (CT7)

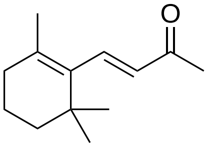

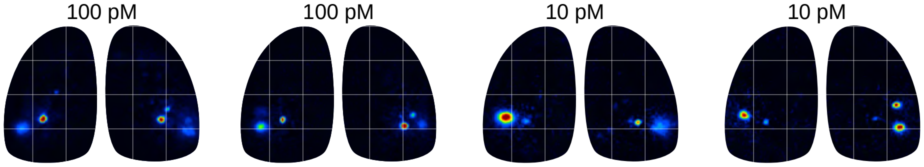

98. β-damascone (CT8)

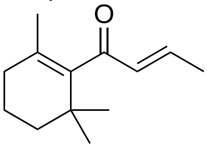

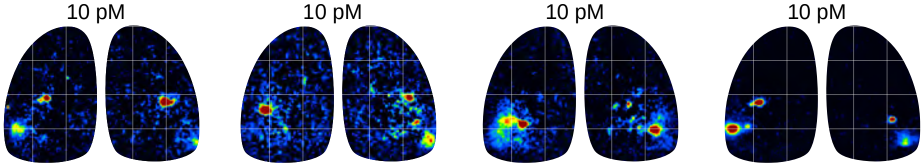

99. Damascenone (CT9)

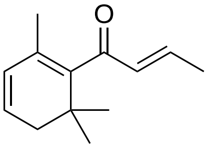

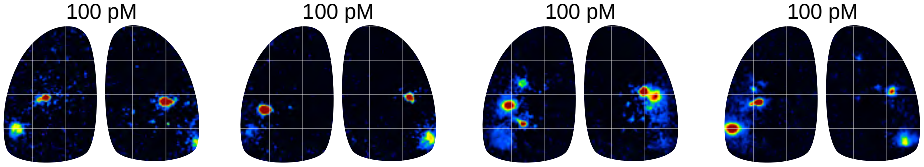

Cyclic Terpenoid II

MOUSE 1 MOUSE 2 MOUSE 3 MOUSE 4

100. Menthone (CT10)

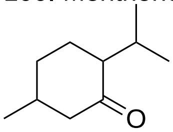

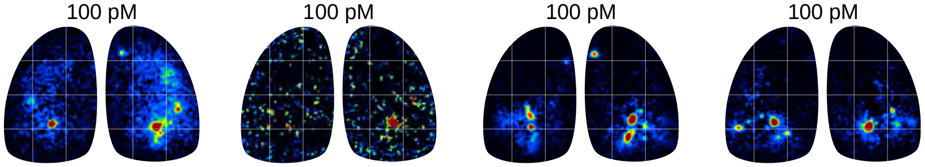

101. (R)-(+)-pulegone (CT11)

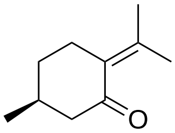

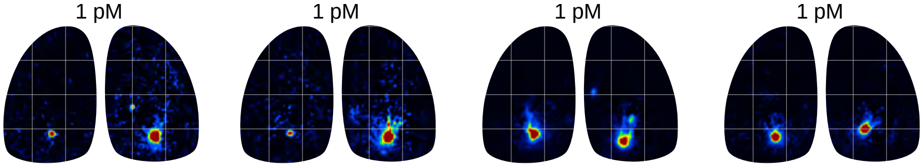

102. (+)-isomethone (CT12)

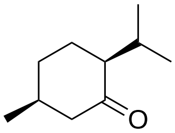

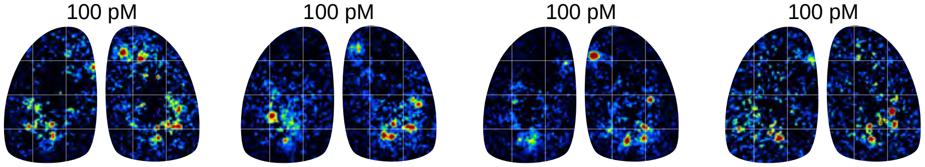

103. Nootkatone (CT13)

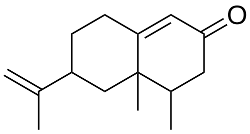

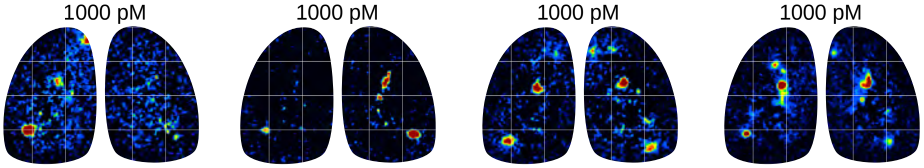

104. (+/-)-menthol (CT14)

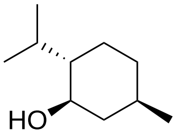

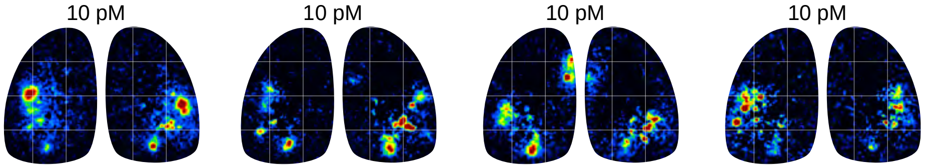

105. (+)-neomenthol (CT15)

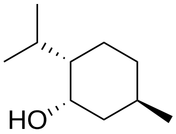

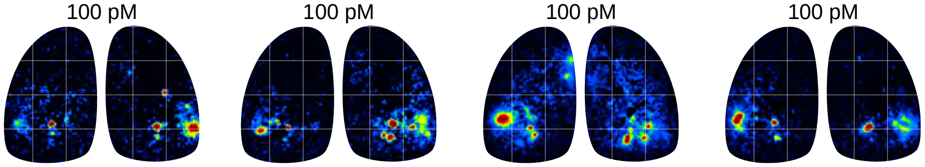

106. (+/-)-geosmin (CT16)

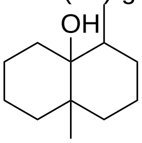

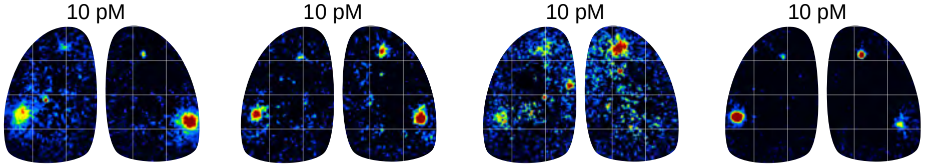

107. (-)-ambroxide (CT17)

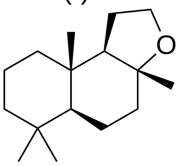

No response at 1000 pM

108. (+)-menthofuran (CT18)

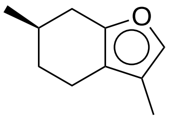

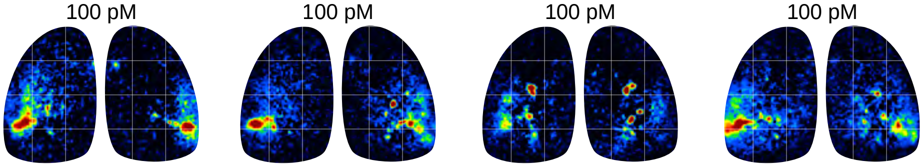

Furan/Pyrone/Lactone

MOUSE 1 MOUSE 2 MOUSE 3 MOUSE 4

109. 2-pentylfuran (F1)

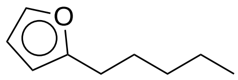

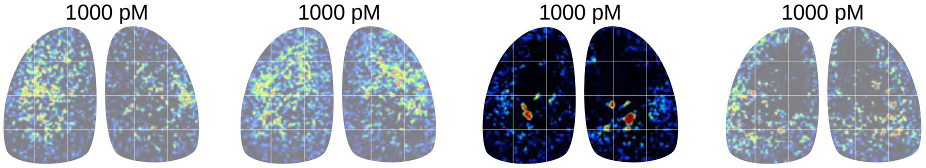

110. 5-ethyl-4-hydroxy-2-methyl-3(2H) furanone (F2)

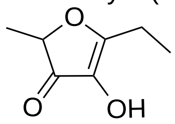

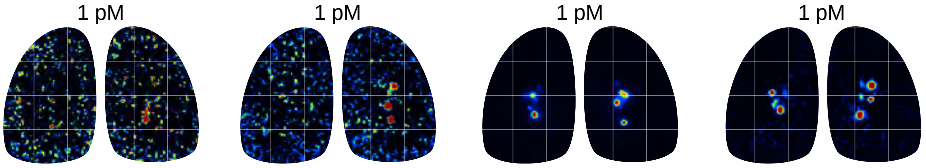

111. 5-methyl furfural (F3)

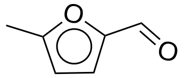

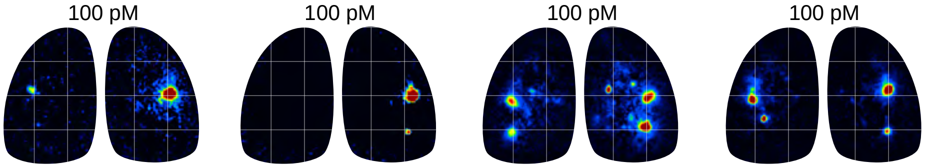

112. Ethyl maltol (F4)

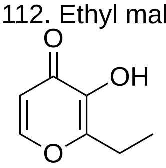

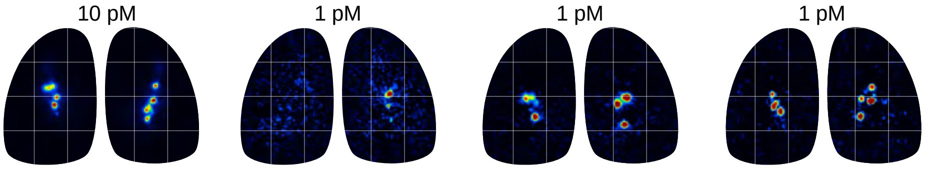

113. γ-undecalactone (F5)

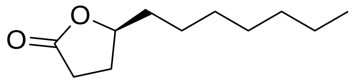

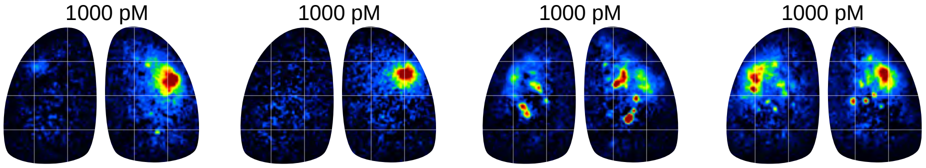

114. Coumarin (F6)

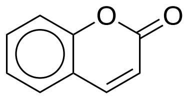

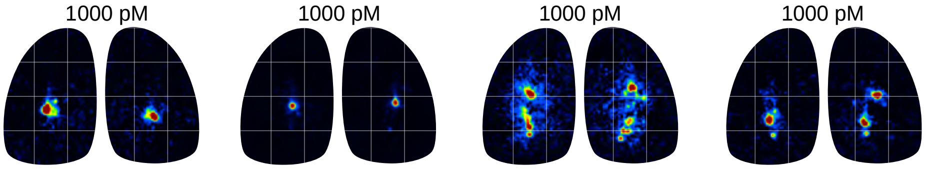

115. 1-furfurylpyrrole (F7)

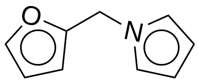

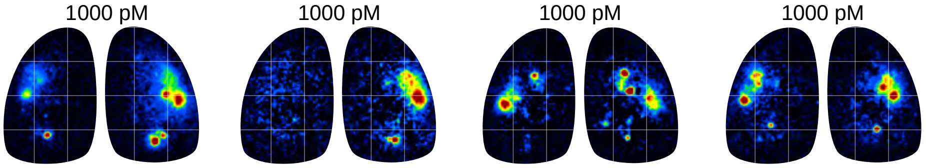

Amine I

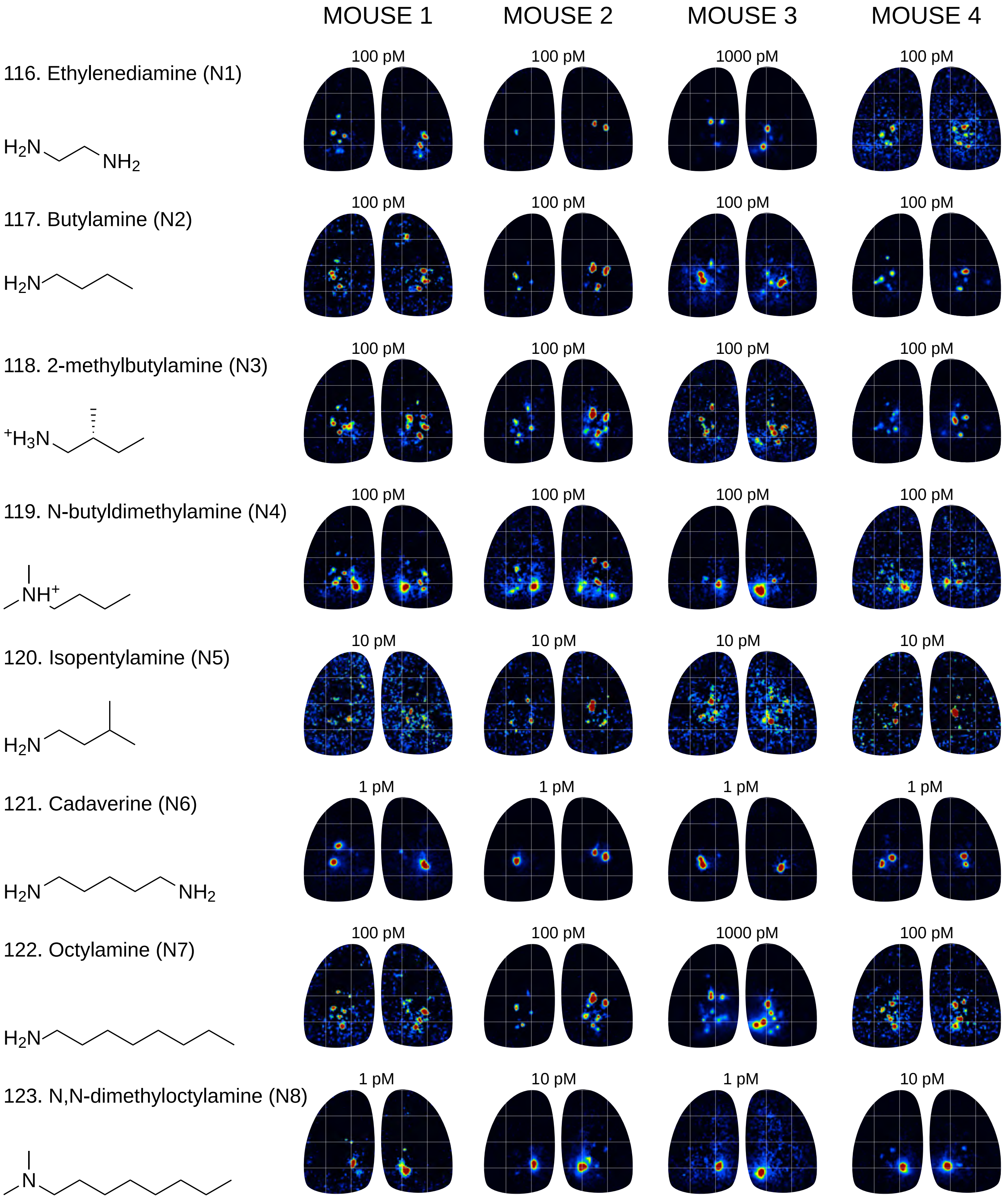

MOUSE 1
MOUSE 2
MOUSE 3
MOUSE 4
116. Ethylenediamine (N1)
100 pM
100 pM
1000 pM
100 pM
117. Butylamine (N2)
100 pM
100 pM
100 pM
100 pM
118. 2-methylbutylamine (N3)
100 pM
100 pM
100 pM
100 pM
119. N-butyldimethylamine (N4)
100 pM
100 pM
100 pM
100 pM
120. Isopentylamine (N5)
10 pM
10 pM
10 pM
10 pM
121. Cadaverine (N6)
1 pM
1 pM
1 pM
1 pM
122. Octylamine (N7)
100 pM
100 pM
1000 pM
100 pM
123. N,N-dimethyloctylamine (N8)
1 pM
10 pM
1 pM
10 pM

Amine II

	MOUSE 1	MOUSE 2	MOUSE 3	MOUSE 4

124. Cyclohexylamine (N9)

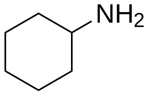

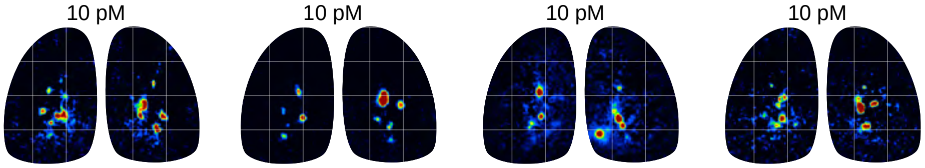

125. N,N-dimethylcyclohexylamine (N10)

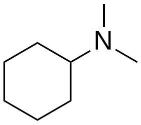

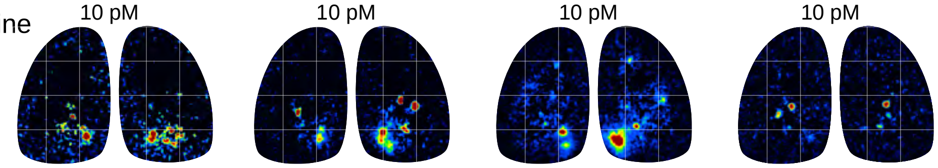

126. Phenethylamine (N11)

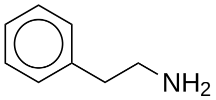

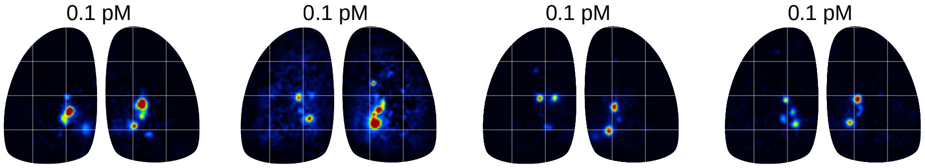

127. Benzylamine (N12)

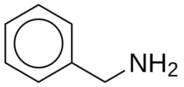

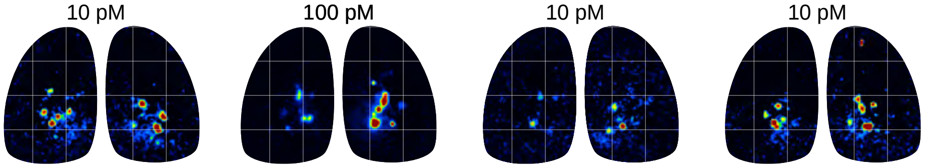

128. Tyramine (N13)

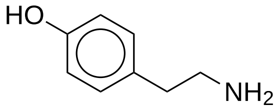

No response at 1000 pM

129. N,N-dimethyl-2-phenethylamine (N14)

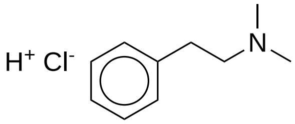

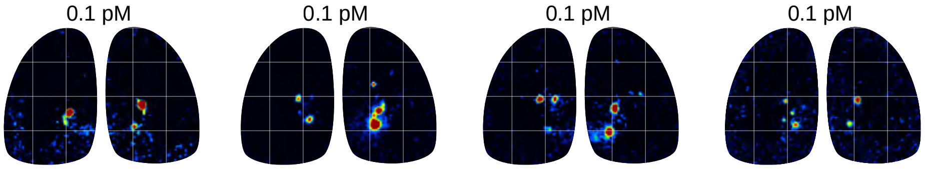

130. 3-phenylpropylamine (N15)

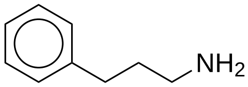

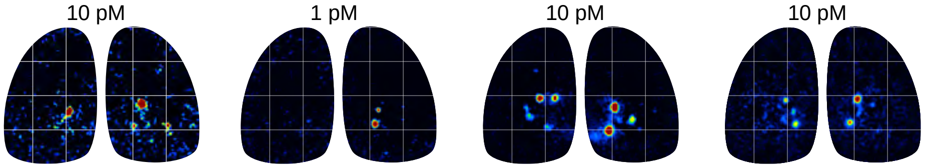

131. N-methyl piperidine (N16)

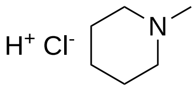

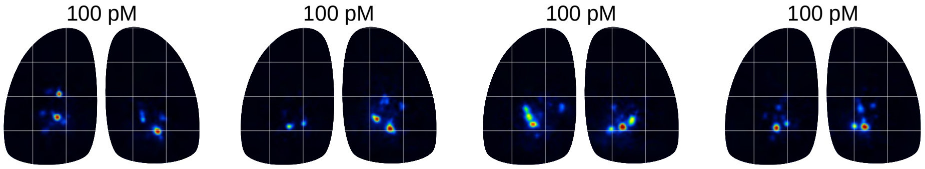

132. 5-methyl heptan-3-one oxime (N17.M8)

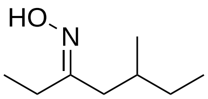

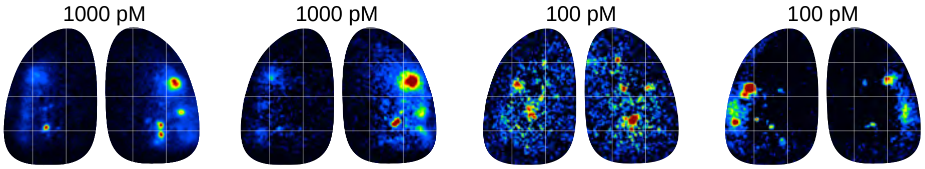

Pyrazine I

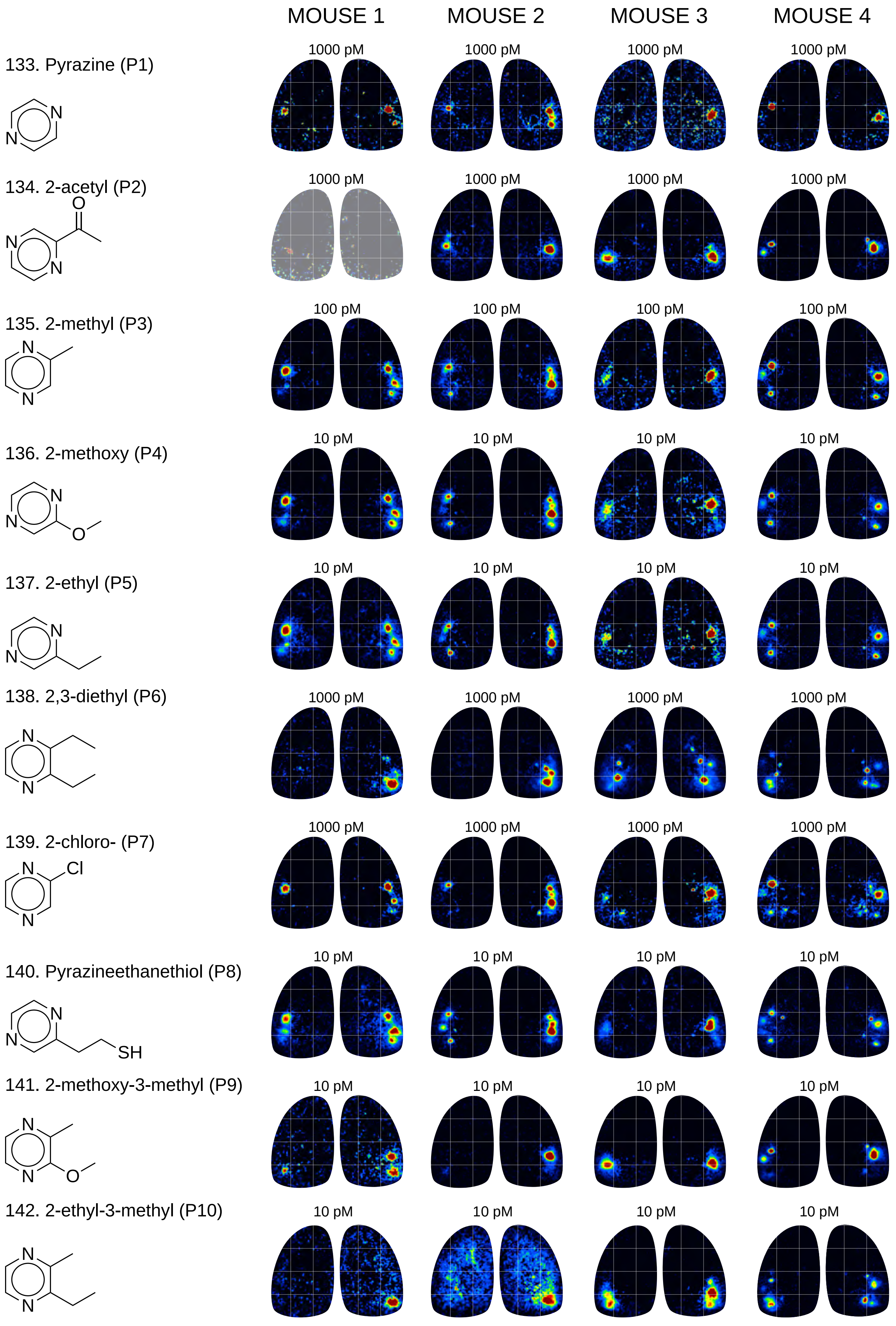
MOUSE 1
MOUSE 2
MOUSE 3
MOUSE 4
133. Pyrazine (P1)
1000 pM 1000 pM 1000 pM 1000 pM
134. 2-acetyl (P2)
1000 pM 1000 pM 1000 pM 1000 pM
135. 2-methyl (P3)
100 pM 100 pM 100 pM 100 pM
136. 2-methoxy (P4)
10 pM 10 pM 10 pM 10 pM
137. 2-ethyl (P5)
10 pM 10 pM 10 pM 10 pM
138. 2,3-diethyl (P6)
1000 pM 1000 pM 1000 pM 1000 pM
139. 2-chloro- (P7)
1000 pM 1000 pM 1000 pM 1000 pM
140. Pyrazineethanethiol (P8)
10 pM 10 pM 10 pM 10 pM
141. 2-methoxy-3-methyl (P9)
10 pM 10 pM 10 pM 10 pM
142. 2-ethyl-3-methyl (P10)
10 pM 10 pM 10 pM 10 pM

Pyrazine II

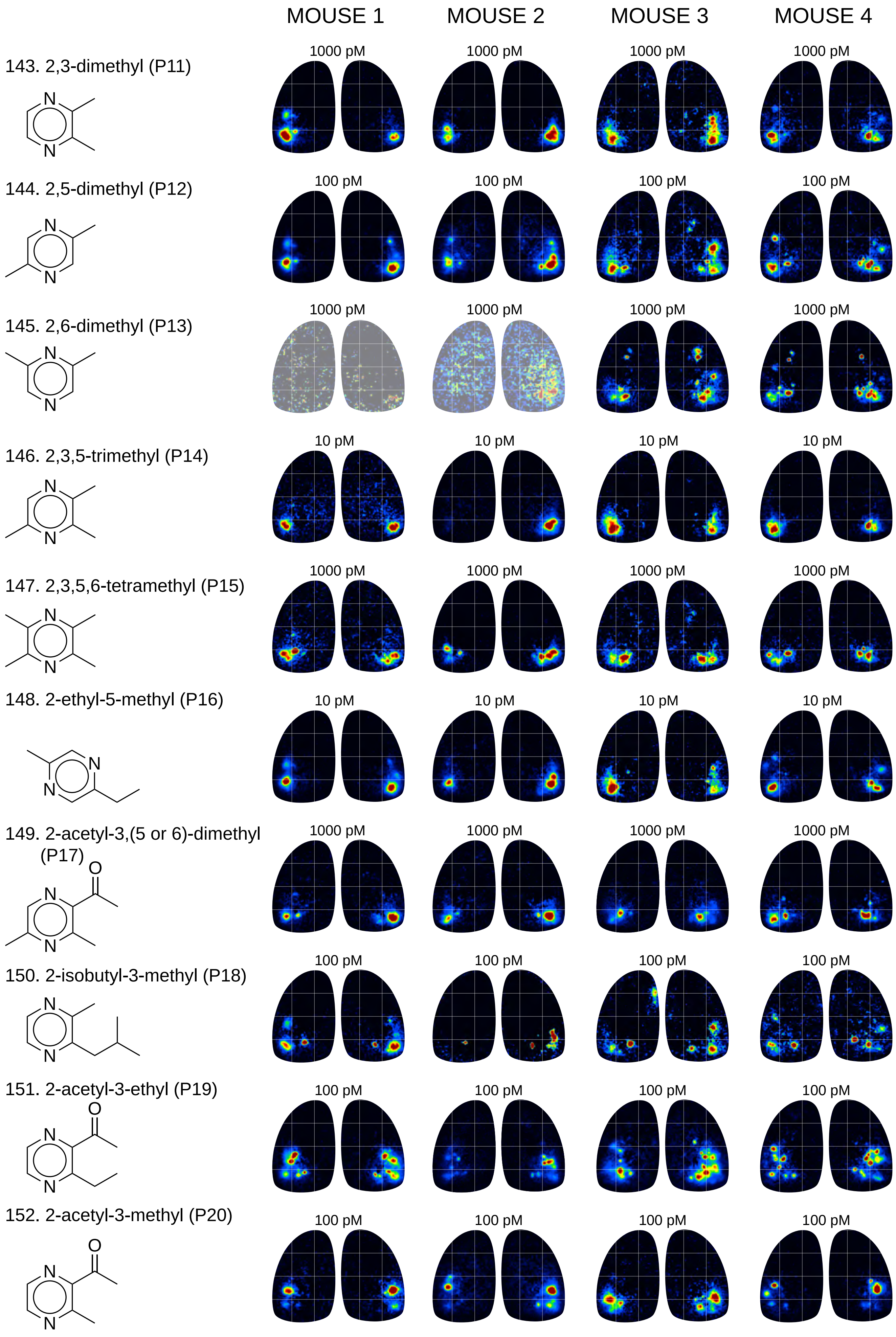
MOUSE 1
MOUSE 2
MOUSE 3
MOUSE 4
143. 2,3-dimethyl (P11)
1000 pM
1000 pM
1000 pM
1000 pM
144. 2,5-dimethyl (P12)
100 pM
100 pM
100 pM
100 pM
145. 2,6-dimethyl (P13)
1000 pM
1000 pM
1000 pM
1000 pM
146. 2,3,5-trimethyl (P14)
10 pM
10 pM
10 pM
10 pM
147. 2,3,5,6-tetramethyl (P15)
1000 pM
1000 pM
1000 pM
1000 pM
148. 2-ethyl-5-methyl (P16)
10 pM
10 pM
10 pM
10 pM
149. 2-acetyl-3,(5 or 6)-dimethyl (P17)
1000 pM
1000 pM
1000 pM
1000 pM
150. 2-isobutyl-3-methyl (P18)
100 pM
100 pM
100 pM
100 pM
151. 2-acetyl-3-ethyl (P19)
100 pM
100 pM
100 pM
100 pM
152. 2-acetyl-3-methyl (P20)
100 pM
100 pM
100 pM
100 pM

Pyrazine III

153. 2-ethyl-3-methoxy (P21)

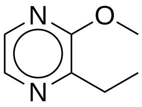

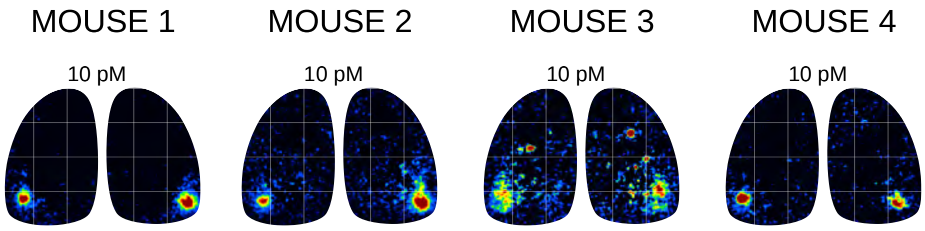

154. 2-methoxy-3(5 or 6)-isopropyl (P22)

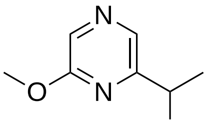

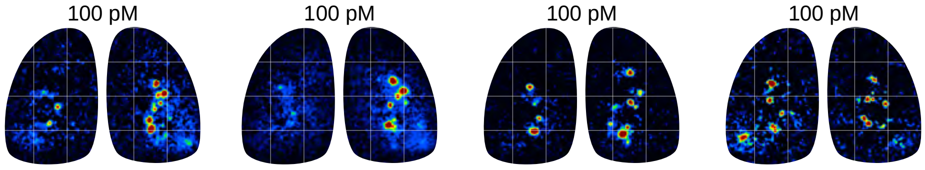

155. 2-isobutyl-3-methoxy (P23)

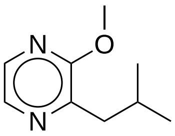

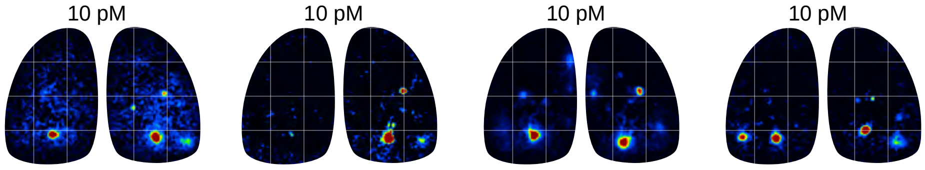

156. 2-methoxy-3-(1-methylpropyl) (P24)

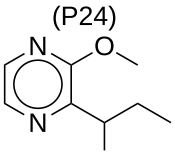

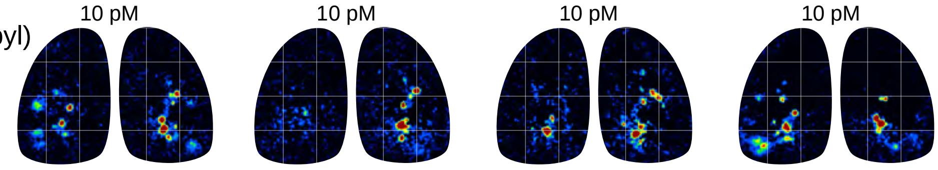

157. 2-isopropyl-3-methoxy (P25)

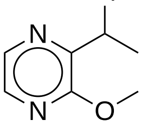

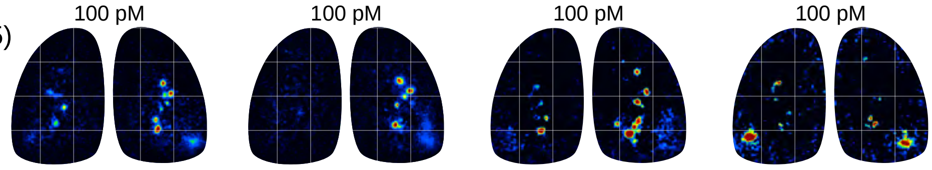

158. 5H-5-methyl-6,7-dihydrocyclopenta[b] (P26)

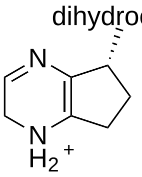

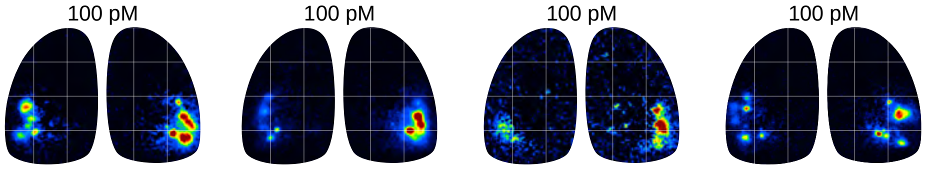

159. 5,6,7,8-tetrahydroquinoxaline (P27)

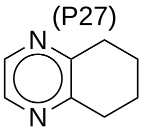

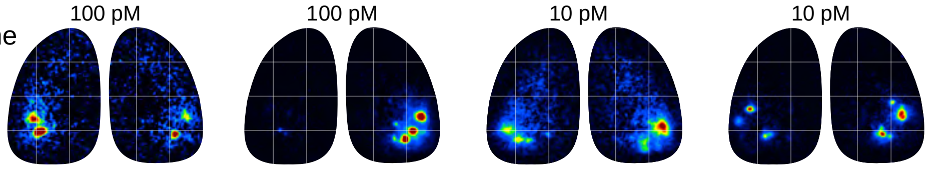

160. 5-methylquinoxaline (P28)

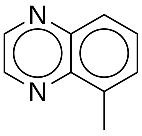

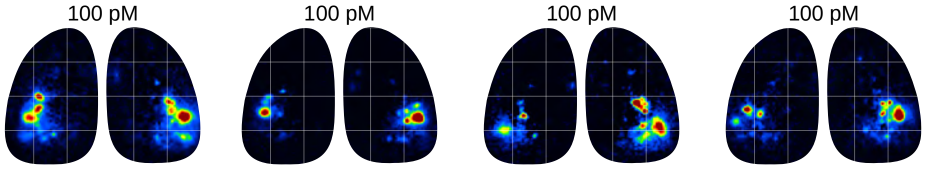

Pyrrole/Pyridine

161. 2-acetylpyridine (NS1)

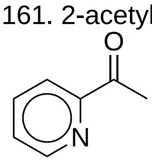

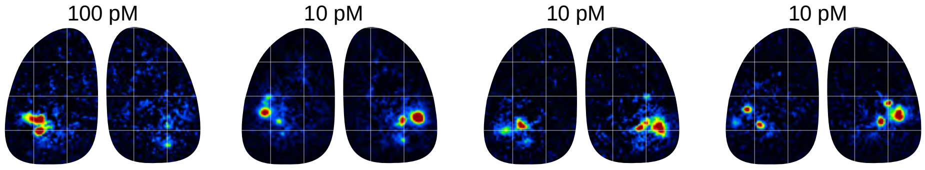

162. 4-tert-butylpyridine (NS2)

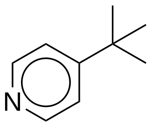

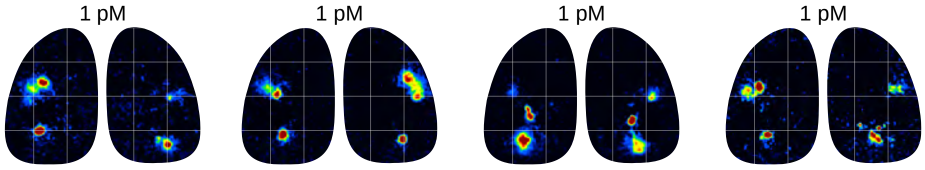

163. Indole (NS3)

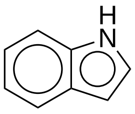

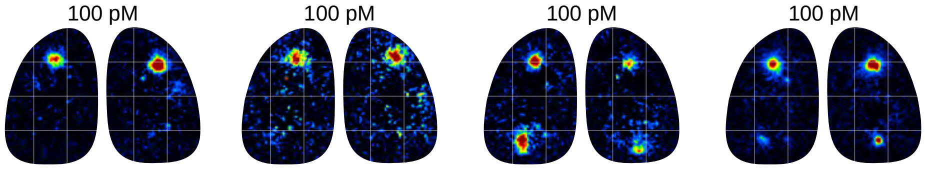

164. 2-methylindole (NS4)

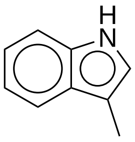

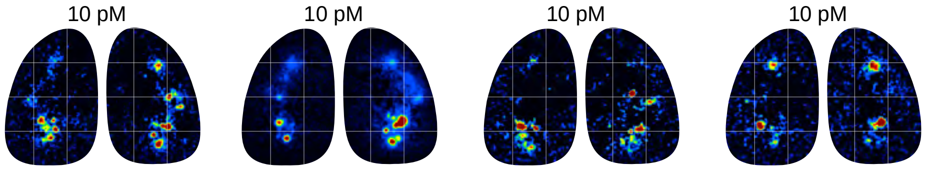

Thiazole/Thiane

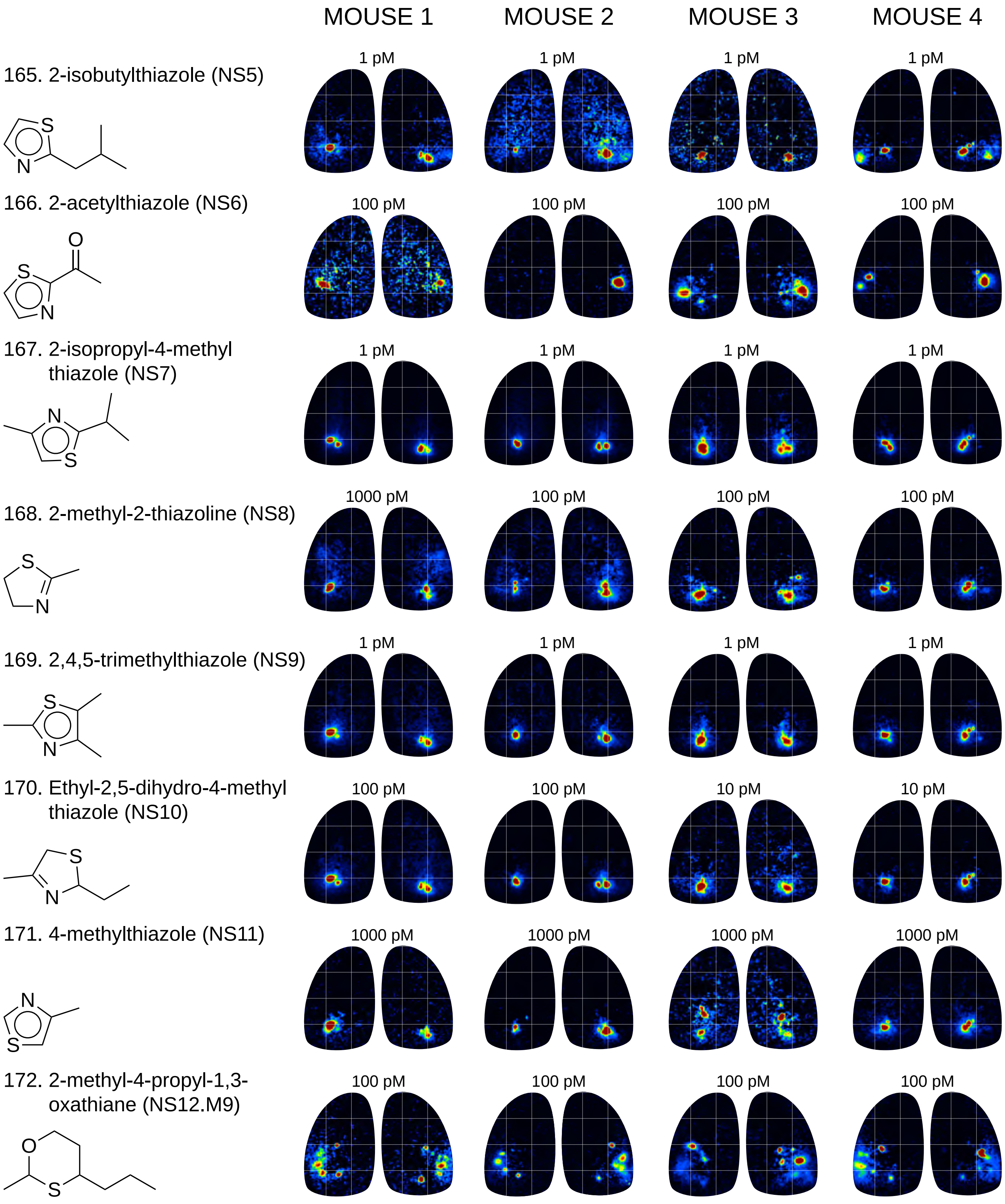

Sulfide/Thiol

MOUSE 1 | MOUSE 2 | MOUSE 3 | MOUSE 4

173. Dimethyl trisulfide (S1)

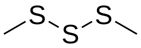

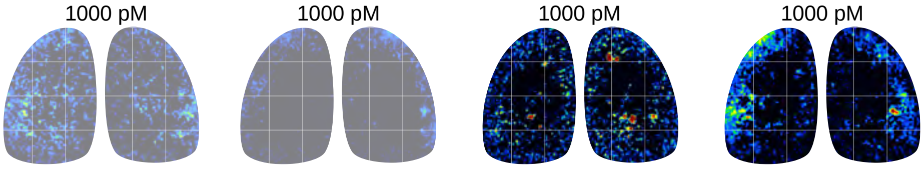

174. Allyl disulfide (S2)

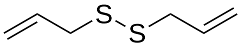

No response at 1000 pM

175. 4-methoxy-2-methyl-2-butanethiol (S3)

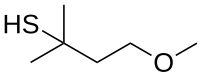

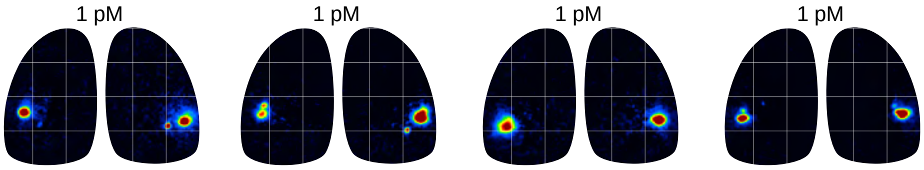

176. Methional (S4.M10)

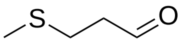

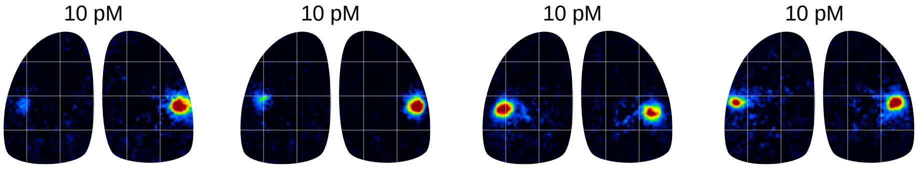

177. 3-mercaptohexyl acetate (S5)

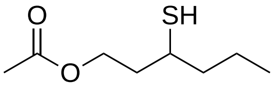

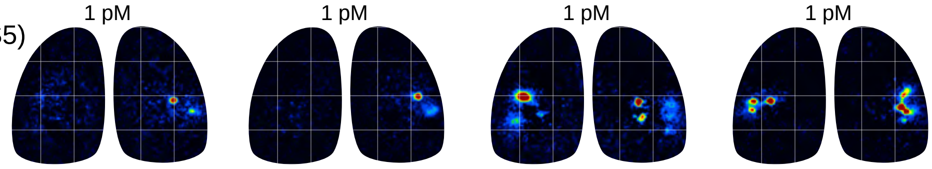

178. 3-(methylthio)-1-hexanol (S6)

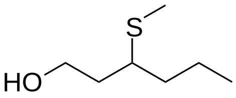

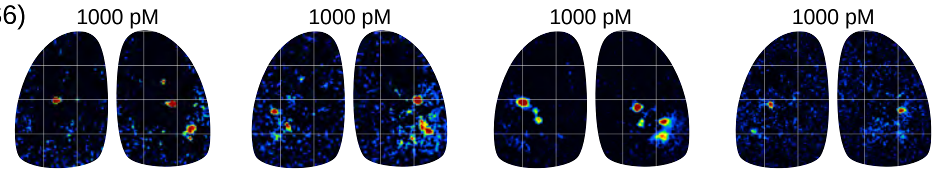

179. Furfuryl mercaptan (S7.M11)

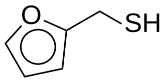

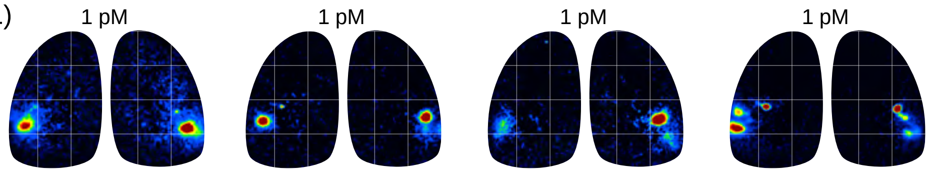

180. Difurfuryl disulfide (S8.M12)

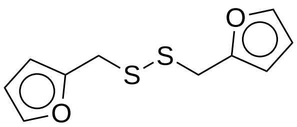

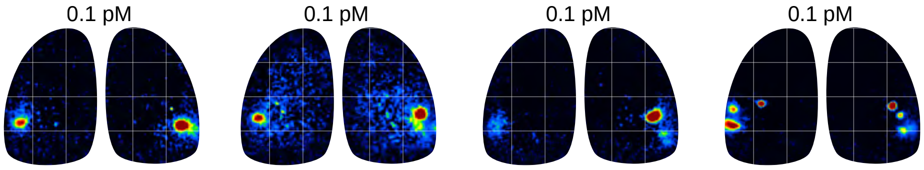

181. Fufrfuryl methyl sulfide (S9.M13)

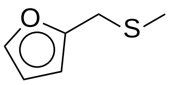

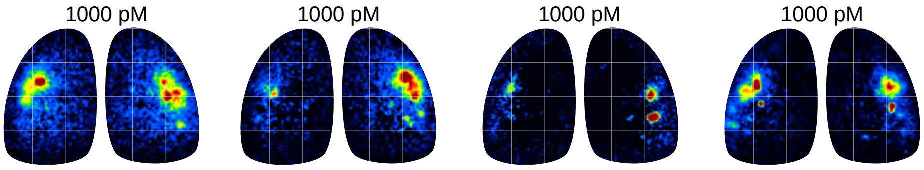

182. 2-methyl-3-tetrahydrofuranthiol (S10)

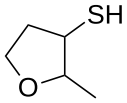

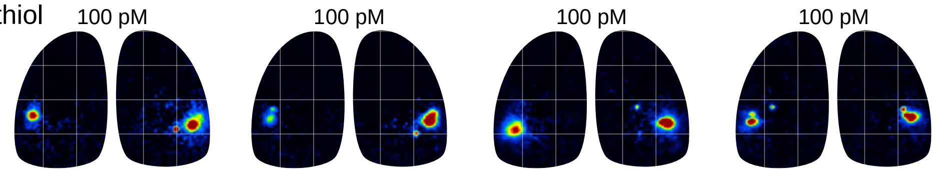

Alkene

MOUSE 1 MOUSE 2 MOUSE 3 MOUSE 4

183. Myrcene (ENE1)

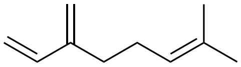

No response at 1000 pM

184. 1,3,5-undecatriene (ENE2)

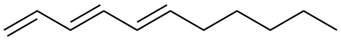

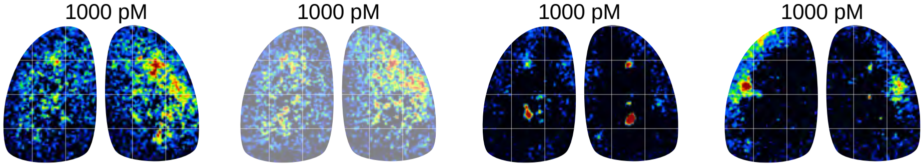

185. (R)-+-limonene (ENE3)

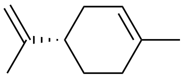

No response at 1000 pM

Supplementary File 2. Functional Atlas of Sensory Inputs to the Dorsal Mouse Olfactory Bulb. Compilation of olfactory sensory neuron response maps to the 185-odorant panel imaged in each of four OMP-IRES-tTA; TetO-GCaMP6s mice. See Methods for details of odorant presentation, imaging and map generation. Each map is scaled to its own maximum (mean of highest 65 pixels) and clipped at $\Delta F=0$. Concentrations are given as estimated delivered concentration to the mouse nose, rounded to the nearest order of magnitude to allow for deviations from ideal behavior of the odorant in its solvent. See Supplementary File 1 for more precise estimates of delivered concentration. Grid overlay is for facilitating visual comparison across maps. Odorants are grouped by major structural features and are numbered in order of presentation in Main figures and Supplementary File 1. Letter-number identifier matches that in Fig. 5A and in Supplementary File 1. Maps with no measured response in any glomerulus of a given mouse are shown at 50% opacity. Odorants failing to evoke a response in all four mice are indicated with text.
