# Supplementary material for: Mapping odorant sensitivities reveals a sparse but structured representation of olfactory chemical space by sensory input to the mouse olfactory bulb
Source: eLife. 2022 Jul 21;11:e80470. doi: 10.7554/eLife.80470 (PMC9352350; doi:10.7554/eLife.80470)
Supplement: Supplementary file 3. — For comparisons to Ma et al., 2012 we used the lowest reported effective dilutions of saturated vapor from the ‘GIA0512’ dataset, available at https://www.pnas.org/doi/full/10.1073/pnas.1117491109#supplementary-materials. Estimated molar concentrations were calculated based on reported vapor pressures, as described in the Materials and methods. [file elife-80470-supp3.docx]

| **Odorant** | **Current study conc. (M)** | **Ma et al., 2012 conc. (M)** | |
| --- | --- | --- | --- |
| butyric acid | 1.6E-11 | 2.4E-07 |  |
| 2-methylbutyric acid | 5.5E-12 | 7.2E-09 |  |
| valeric acid | 5.5E-12 | 2.9E-09 |  |
| heptanoic acid | 8.9E-11 | 1.6E-08 |  |
| butyraldehyde | 6.0E-09 | 1.4E-06 |  |
| 2-methylbutyraldehyde | 7.8E-11 | 1.5E-07 |  |
| trans-2-methyl-2-butenal | 1.3E-11 | 2.6E-07 |  |
| valeraldehyde | 1.9E-09 | 3.7E-07 |  |
| isovaleraldehyde | 3.7E-09 | 6.9E-07 |  |
| hexanal | 7.2E-10 | 1.6E-07 |  |
| heptanal | 2.1E-09 | 5.2E-08 |  |
| octanal | 1.1E-09 | 1.7E-08 |  |
| butyl acetate | 8.9E-10 | 1.7E-06 |  |
| isoamyl acetate | 4.3E-10 | 8.2E-08 |  |
| ethyl butyrate | 1.1E-09 | 1.8E-07 |  |
| vinyl butyrate | 9.1E-11 | 1.7E-07 |  |
| methyl valerate | 8.4E-10 | 2.7E-07 |  |
| ethyl tiglate | 2.3E-12 | 4.4E-08 |  |
| isopropyl tiglate | 2.1E-11 | 4.1E-08 |  |
| 2-butanone | 6.7E-10 | 1.2E-05 |  |
| 2-pentanone | 2.9E-09 | 5.0E-07 |  |
| 2-hexanone | 1.0E-09 | 1.7E-07 |  |
| 2-octanone | 1.4E-10 | 2.0E-07 |  |
| 2-nonanone | 4.9E-10 | 9.2E-07 |  |
| butylamine | 6.3E-10 | 1.2E-04 |  |
| isopentylamine | 3.2E-11 | 6.2E-07 |  |
| cyclohexylamine | 6.3E-11 | 1.5E-05 |  |
| benzaldehyde | 7.7E-11 | 1.9E-08 |  |
| acetophenone | 1.4E-11 | 5.8E-08 |  |
| 4-methylanisole | 9.1E-11 | 1.8E-08 |  |
| 2-isobutylthiazole | 3.0E-12 | 2.7E-09 |  |

**Supplementary File 3. Table of odorants and estimated odorant concentrations used in prior study comparison.**

For comparisons to (Ma et al., 2012) we used the lowest reported effective dilutions of saturated vapor from the ‘GIA0512’ dataset, available at https://www.pnas.org/doi/full/10.1073/pnas.1117491109#supplementary-materials. Estimated molar concentrations were calculated based on reported vapor pressures, as described in the Methods.
