# Supplementary material for: Mapping odorant sensitivities reveals a sparse but structured representation of olfactory chemical space by sensory input to the mouse olfactory bulb
Source: eLife. 2022 Jul 21;11:e80470. doi: 10.7554/eLife.80470 (PMC9352350; doi:10.7554/eLife.80470)
Supplement: Supplementary file 4. — Compilation of diagnostic odorants and example response maps for each of the 26 functionally-identified glomeruli (see Text and Table 1). Concentrations are given as estimated delivered concentration, rounded to the nearest order of magnitude. See Supplementary file 1 for more precise estimates of delivered concentration. Bar plots indicate relative response magnitudes evoked by all effective odorants for each glomerulus, taken from the median response spectrum across the eight OBs. Positions of each glomerulus are shown in lower right of p. 1, reproduced from Figure 2. [file elife-80470-supp4.pdf]

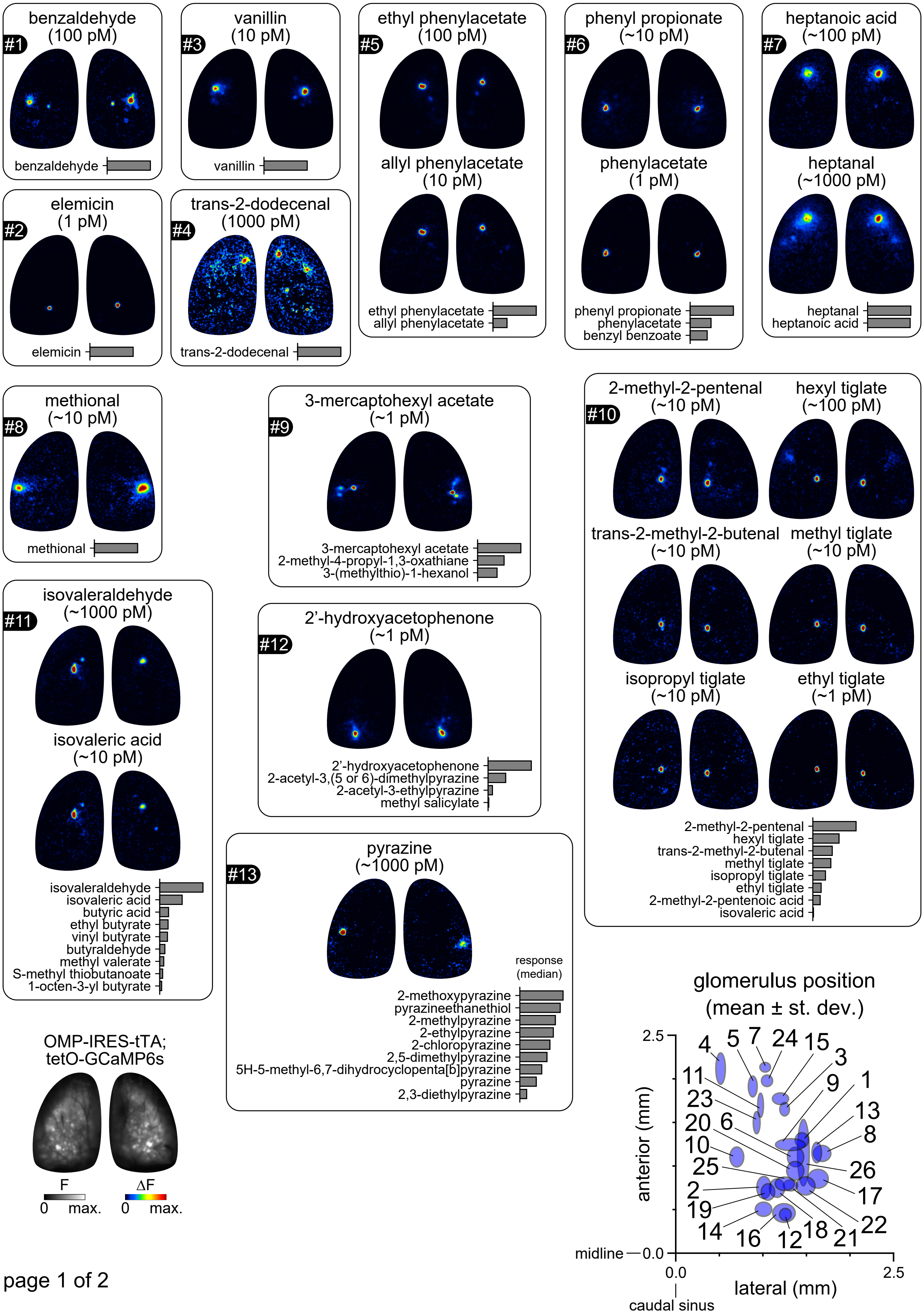

benzaldehyde (100 pM)
#1
benzaldehyde
vanillin (10 pM)
#3
vanillin
ethyl phenylacetate (100 pM)
#5
allyl phenylacetate (10 pM)
ethyl phenylacetate
allyl phenylacetate
phenyl propionate (~10 pM)
#6
phenylacetate (1 pM)
phenyl propionate
phenylacetate
benzyl benzoate
heptanoic acid (~100 pM)
#7
heptanal (~1000 pM)
heptanal
heptanoic acid
elemicin (1 pM)
#2
elemicin
trans-2-dodecenal (1000 pM)
#4
trans-2-dodecenal
methional (~10 pM)
#8
methional
3-mercaptohexyl acetate (~1 pM)
#9
3-mercaptohexyl acetate
2-methyl-4-propyl-1,3-oxathiane
3-(methylthio)-1-hexanol
2-methyl-2-pentenal (~10 pM)
hexyl tiglate (~100 pM)
#10
trans-2-methyl-2-butenal (~10 pM)
methyl tiglate (~10 pM)
isopropyl tiglate (~10 pM)
ethyl tiglate (~1 pM)
2-methyl-2-pentenal
hexyl tiglate
trans-2-methyl-2-butenal
methyl tiglate
isopropyl tiglate
ethyl tiglate
2-methyl-2-pentenoic acid
isovaleric acid
isovaleraldehyde (~1000 pM)
#11
isovaleric acid (~10 pM)
isovaleraldehyde
isovaleric acid
butyric acid
ethyl butyrate
vinyl butyrate
butyraldehyde
methyl valerate
S-methyl thiobutanoate
1-octen-3-yl butyrate
2'-hydroxyacetophenone (~1 pM)
#12
2'-hydroxyacetophenone
2-acetyl-3,(5 or 6)-dimethylpyrazine
2-acetyl-3-ethylpyrazine
methyl salicylate
pyrazine (~1000 pM)
#13
response (median)
2-methoxypyrazine
pyrazineethanethiol
2-methylpyrazine
2-ethylpyrazine
2-chloropyrazine
2,5-dimethylpyrazine
5H-5-methyl-6,7-dihydrocyclopenta[b]pyrazine
pyrazine
2,3-diethylpyrazine
OMP-IRES-tTA; tetO-GCaMP6s
F
ΔF
0 max.
0 max.
glomerulus position (mean ± st. dev.)
2.5
anterior (mm)
midline
0.0
0.0
2.5
lateral (mm)
caudal sinus
4 5 7 24 15 3 11 9 1 23 13 20 6 8 10 25 26 2 17 19 22 14 16 12 18 21

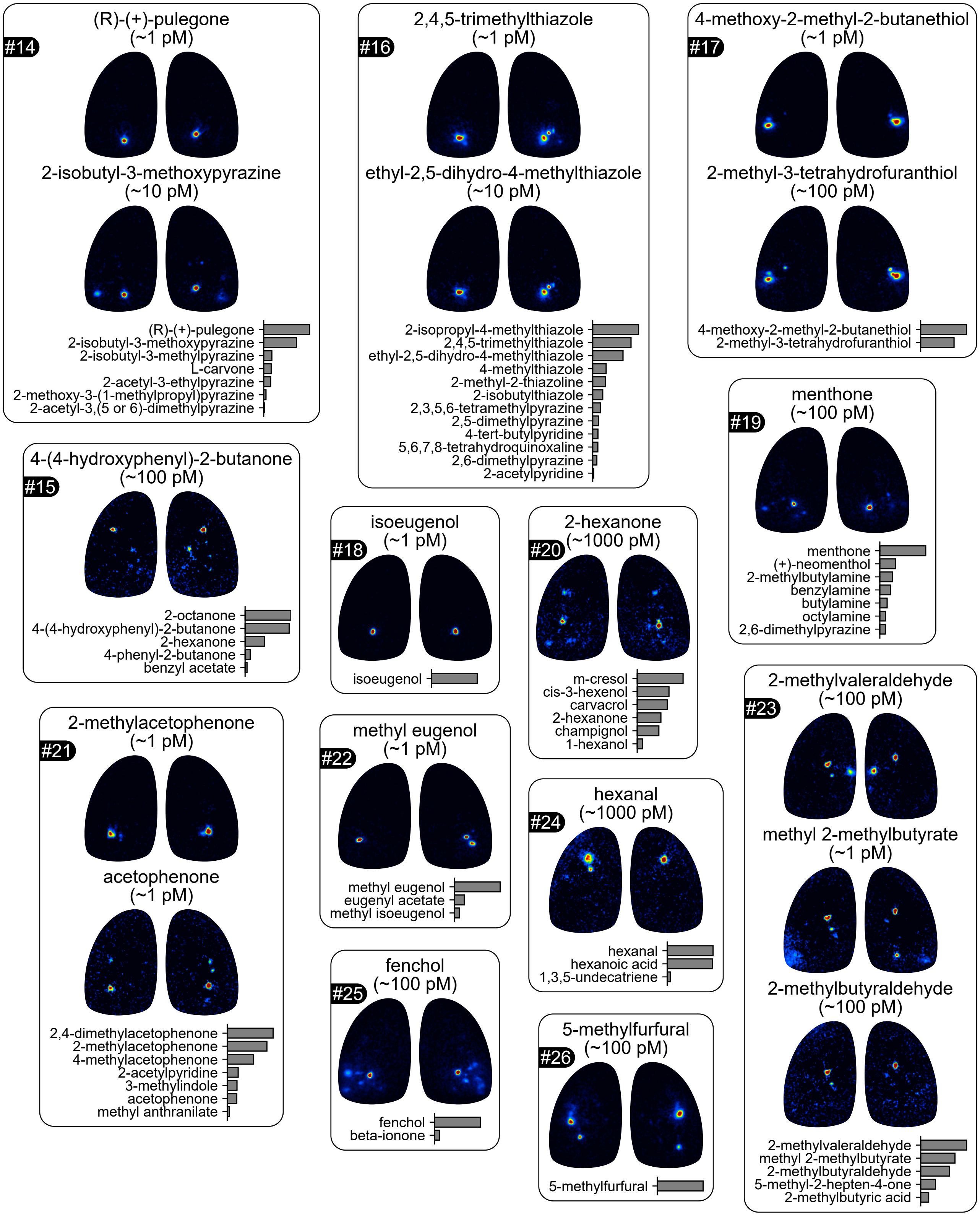

#14
(R)-(+)-pulegone (~1 pM)
2-isobutyl-3-methoxypyrazine (~10 pM)
(R)-(+)-pulegone
2-isobutyl-3-methoxypyrazine
2-isobutyl-3-methylpyrazine
L-carvone
2-acetyl-3-ethylpyrazine
2-methoxy-3-(1-methylpropyl)pyrazine
2-acetyl-3,(5 or 6)-dimethylpyrazine
#16
2,4,5-trimethylthiazole (~1 pM)
ethyl-2,5-dihydro-4-methylthiazole (~10 pM)
2-isopropyl-4-methylthiazole
2,4,5-trimethylthiazole
ethyl-2,5-dihydro-4-methylthiazole
4-methylthiazole
2-methyl-2-thiazoline
2-isobutylthiazole
2,3,5,6-tetramethylpyrazine
2,5-dimethylpyrazine
4-tert-butylpyridine
5,6,7,8-tetrahydroquinoxaline
2,6-dimethylpyrazine
2-acetylpyridine
#17
4-methoxy-2-methyl-2-butanethiol (~1 pM)
2-methyl-3-tetrahydrofuranthiol (~100 pM)
4-methoxy-2-methyl-2-butanethiol
2-methyl-3-tetrahydrofuranthiol
#15
4-(4-hydroxyphenyl)-2-butanone (~100 pM)
2-octanone
4-(4-hydroxyphenyl)-2-butanone
2-hexanone
4-phenyl-2-butanone
benzyl acetate
#18
isoeugenol (~1 pM)
isoeugenol
#20
2-hexanone (~1000 pM)
m-cresol
cis-3-hexenol
carvacrol
2-hexanone
champignol
1-hexanol
#19
menthone (~100 pM)
menthone
(+)-neomenthol
2-methylbutylamine
benzylamine
butylamine
octylamine
2,6-dimethylpyrazine
#21
2-methylacetophenone (~1 pM)
acetophenone (~1 pM)
2,4-dimethylacetophenone
2-methylacetophenone
4-methylacetophenone
2-acetylpyridine
3-methylindole
acetophenone
methyl anthranilate
#22
methyl eugenol (~1 pM)
methyl eugenol
eugenyl acetate
methyl isoeugenol
#24
hexanal (~1000 pM)
hexanal
hexanoic acid
1,3,5-undecatriene
#23
2-methylvaleraldehyde (~100 pM)
methyl 2-methylbutyrate (~1 pM)
2-methylbutyraldehyde (~100 pM)
2-methylvaleraldehyde
methyl 2-methylbutyrate
2-methylbutyraldehyde
5-methyl-2-hepten-4-one
2-methylbutyric acid
#25
fenchol (~100 pM)
fenchol
beta-ionone
#26
5-methylfurfural (~100 pM)
5-methylfurfural

Supplementary File 4. Atlas of Functionally Identified Glomeruli.

Compilation of diagnostic odorants and example response maps for each of the 26 functionally-identified glomeruli (see Text and Table 1). Concentrations are given as estimated delivered concentration, rounded to the nearest order of magnitude. See Supplementary File 1 for more precise estimates of delivered concentration. Bar plots indicate relative response magnitudes evoked by all effective odorants for each glomerulus, taken from the median response spectrum across the 8 OBs. Positions of each glomerulus are shown in lower right of p. 1, reproduced from Fig. 2.
