# Supplementary material for: Mapping odorant sensitivities reveals a sparse but structured representation of olfactory chemical space by sensory input to the mouse olfactory bulb
Source: eLife. 2022 Jul 21;11:e80470. doi: 10.7554/eLife.80470 (PMC9352350; doi:10.7554/eLife.80470)
Supplement: Supplementary file 5. — See Materials and methods and Table 1 for definition of Error ratio and Median ORS corr. [file elife-80470-supp5.docx]

|  | **Odorant** | **Est. conc. (M)** | **Error**  **ratio** | **Median**  **ORS corr.** |
| --- | --- | --- | --- | --- |
| - | 2,3,5-trimethylpyrazine | 1E-11 | 0.12 | 0.74 |
| - | p-anisaldehyde | 8E-12 | 0.21 | 0.93 |
| - | piperonal | 2E-12 | 0.21 | 0.93 |
| - | cadaverine | 2E-12 | 0.23 | 0.98 |
| - | furfuryl mercaptan | 3E-12 | 0.24 | 0.92 |
| - | difurfuryl disulfide | 4E-13 | 0.24 | 0.92 |
| - | 2-methyl-2-pentenoic acid | 4E-14 | 0.25 | 0.93 |
| - | 2-methylpyrazine | 7E-11 | 0.26 | 0.85 |
| - | 2-chloropyrazine | 2E-9 | 0.26 | 0.85 |
| - | damascenone | 2E-10 | 0.29 | 0.76 |
| - | N,N-dimethyloctylamine | 3E-11 | 0.32 | 1.00 |
| - | beta-damascone | 1E-11 | 0.32 | 0.81 |
| - | 2-ethyl-5-methylpyrazine | 1E-11 | 0.36 | 0.74 |
| - | 3-(methylthio)-1-hexanol | 6E-9 | 0.39 | 0.89 |
| - | benzyl benzoate | 4E-11 | 0.39 | 0.82 |
| - | 4-methylthiazole | 8E-10 | 0.39 | 0.87 |
| - | eugenol | 7E-13 | 0.40 | 0.59 |
| - | 2-methylbutyric acid | 5E-12 | 0.43 | 0.95 |
| - | beta-ionone | 7E-11 | 0.43 | 0.73 |
| - | L-carvone | 1E-10 | 0.43 | 0.66 |
| - | 2-methoxy-3-methylpyrazine | 5E-11 | 0.45 | 0.88 |
| - | 4-methylacetophenone | 7E-12 | 0.48 | 0.81 |
| - | N,N-dimethyl-2-phenethylamine | 5E-13 | 0.50 | 0.75 |
| - | N-methyl piperidine | 2E-10 | 0.50 | 0.99 |
| - | 1,3,5-undecatriene | 5E-9 | 0.50 | 0.55 |
| - | 2-methyl-2-thiazoline | 1E-9 | 0.52 | 0.83 |
| - | 4-methoxyacetophenone | 5E-13 | 0.54 | 0.83 |
| - | 2,6-dimethoxyphenol | 2E-12 | 0.54 | 1.00 |
| - | isopentylamine | 3E-11 | 0.55 | 0.67 |
| - | nootkatone | 9E-10 | 0.57 | 0.97 |
| - | champignol | 4E-10 | 0.59 | 0.50 |
| - | 2-acetyl-3,(5 or 6)-dimethylpyrazine | 2E-9 | 0.59 | 0.42 |
| - | (+)-menthofuran | 2E-10 | 0.59 | 0.61 |
| - | 2-ethyl-3-methoxypyrazine | 4E-11 | 0.61 | 0.51 |
| - | 2-octanone | 1E-10 | 0.66 | 0.62 |
| - | furfuryl methyl sulfide | 1E-9 | 0.67 | 0.20 |
| - | geraniol | 1E-9 | 0.67 | 0.29 |
| - | butyrophenone | 2E-11 | 0.70 | 0.50 |
| - | 4-methylanisole | 9E-11 | 0.75 | 0.00 |
| - | benzyl acetate | 1E-10 | 0.86 | 0.15 |

**Supplementary File 5. Additional odorants and concentrations eliciting consistently sparse activation but failing conservative requirements for functional identification.**

See Methods and Table 1 for definition of Error ratio and Median ORS corr.
